# Paralogs of Common Carp Granulocyte Colony-Stimulating Factor (G-CSF) Have Different Functions Regarding Development, Trafficking and Activation of Neutrophils

**DOI:** 10.3389/fimmu.2019.00255

**Published:** 2019-02-19

**Authors:** Fumihiko Katakura, Kohei Nishiya, Annelieke S. Wentzel, Erika Hino, Jiro Miyamae, Masaharu Okano, Geert F. Wiegertjes, Tadaaki Moritomo

**Affiliations:** ^1^Laboratory of Comparative Immunology, Department of Veterinary Medicine, Nihon University, Fujisawa, Japan; ^2^Cell Biology and Immunology Group, Wageningen University & Research, Wageningen, Netherlands; ^3^Aquaculture and Fisheries Group, Wageningen Institute of Animal Science, Wageningen University & Research, Wageningen, Netherlands

**Keywords:** teleost, granulocyte colony-stimulating factor, neutrophil, hematopoiesis, cell migration, respiratory burst

## Abstract

Mammalian granulocyte colony-stimulating factor (G-CSF; CSF3) is a primary cytokine that promotes the development, mobilization, and activation of neutrophils and their precursors. Teleosts have been reported to possess two paralogs as a likely result of the teleost-wide whole genome duplication (WGD) event, but functional divergence of G-CSF paralogs remains poorly understood. Common carp are an allotetraploid species owing to an additional WGD event in the carp lineage and here, we report on genomic synteny, sequence similarity, and phylogeny of four common carp G-CSF paralogs (*g-csfa1* and *g-csfa2*; *g-csfb1* and *g-csfb2*). *G-csfa1* and *g-csfa2* show differential and relatively high gene expression levels, while *g-csfb1* and *g-csfb2* show low basal gene expression levels in most tissues. All paralogs are expressed higher in macrophages than in other leukocyte sub-types and are highly up-regulated by treatment of macrophages with mitogens. Recombinant G-CSFa1 and G-CSFb1 both promoted the proliferation of kidney hematopoietic cells, while only G-CSFb1 induced the differentiation of kidney cells along the neutrophil-lineage. Colony-forming unit assays revealed that G-CSFb1 alone stimulates the formation of CFU-G colonies from head- and trunk-kidney whereas the combination of G-CSFa1 and G-CSFb1 stimulates the formation of both CFU-G and CFU-GM colonies. Recombinant G-CSFa1 and G-CSFb1 also exhibit chemotactic activity against kidney neutrophils and up-regulation of *cxcr1* mRNA expression was highest in neutrophils after G-CSFb1 stimulation. Furthermore, G-CSFb1 more than G-CSFa1 induced priming of kidney neutrophils through up-regulation of a NADPH-oxidase component p47^*phox*^. *In vivo* administration of G-CSF paralogs increased the number of circulating blood neutrophils of carp. Our findings demonstrate that gene duplications in teleosts can lead to functional divergence between paralogs and shed light on the sub-functionalization of G-CSF paralogs in cyprinid fish.

## Introduction

Granulocyte colony-stimulating factor (G-CSF), also called colony-stimulating factor 3 (CSF3), is a primary cytokine that promotes the proliferation, differentiation and survival of neutrophil progenitors and enhances trafficking and immunological functions of mature neutrophils in mammals (1). Human G-CSF is produced mainly by monocytes and macrophages (2), but is also produced by fibroblasts (3), endothelial cells (4), and bone marrow stromal cells (5). Although healthy individuals express low G-CSF protein levels in serum, remarkable elevations of G-CSF production can be induced by several inflammatory stimuli, including increased presence of pro-inflammatory cytokines and LPS during infections (6–8). Effects are mediated by the binding of G-CSF with its cognate receptor G-CSFR on neutrophils and their progenitors, activating downstream signaling cascades and thereby influencing subsequent gene expression and cellular immune responses [reviewed in (1)]. Mice lacking G-CSF/G-CSFR signaling (*G-csf*-deficient or *G-csfr*-deficient mice) exhibit a reduction in myeloid progenitors and impaired neutrophil mobilization into the circulation, resulting in chronic neutropenia (9, 10). This suggests that G-CSF is a major regulator of neutrophil development and contributes to the regulation of multipotent hematopoietic progenitors. At the same time, G-CSF also influences the phenotype and function of mature neutrophils and does so by modulating expression of for example chemokine receptors, up-regulating phagocytosis and production of reactive oxygen species (ROS) and enhancing bactericidal activity of neutrophils (11).

G-CSF was first purified and characterized in mice (12), only later followed by studies in non-mammalian vertebrates such as chicken (13), African-clawed frog (14), and a number of teleost fish species including flounder, fugu, green-spotted pufferfish (13), black rockfish (15), and zebrafish (16). Owing to a teleost-specific whole genome duplication (WGD) event (17), teleost can generally be expected to express two G-CSF paralogs, type A (G-CSFa) and type B (G-CSFb), which may not necessarily have the same function. Indeed, zebrafish express an A and B paralog and earlier studies suggest that both G-CSFa and G-CSFb are required for hematopoietic stem cell (HSC) emergence and expansion of primitive and mature neutrophils and macrophages *in vivo* and *in vitro* (16). *G-csfr* morphants were affected on early myeloid cell migration and development, but had functionally normal myeloid cells (18). Zebrafish G-CSFb was involved in neutrophil mobilization toward an injury site (19), but the contribution of G-CSFa remained unclear. Therefore, the exact role of teleost G-CSF paralogs as regulators of diverse markers of neutrophil activation and/or regulators of multipotent hematopoietic progenitor development has remained unresolved.

In this study, we report on the molecular and functional characterization of G-CSF paralogs from the common carp. The close kinship of zebrafish and carp (20) allows for comparative use of genetic information from the well-described zebrafish genome whereas the large size of carp allowed us to perform cell type specific gene expression and *ex vivo* functional studies on large number of cells. Because common carp is an allotetraploid species owing to an additional WGD event in the carp lineage (21), we report on the cloning and molecular characterization of two type A copies (*g-csfa1* and *g-csfa2*) and two type B copies (*g-csfb1* and *g-csfb2*). For functional characterization we chose to produce recombinant proteins for two G-CSF paralogs particularly highly expressed in macrophages (G-CSFa1 and G-CSFb1) and examined their ability to drive proliferation, differentiation and colony-formation of carp hematopoietic kidney cells along the neutrophil lineage. We also studied the effect of these G-CSF paralogs on neutrophil function including phenotype, chemotaxis and production of ROS. *In vivo* effects of G-CSF paralogs on circulating blood neutrophils were further investigated. We discuss the functions of teleost G-CSF regarding development, trafficking and activation of neutrophils and discuss the importance of studying paralogs of granulocyte colony-stimulating factor.

## Materials and Methods

### Animals

Common Carp (*Cyprinus carpio* L.) were kept at Nihon University (NU) and at Wageningen University (WU). Carp weighing 40–100 g (10 to 15 cm in length) were purchased from commercial farms and reared at NU, Japan. Fish were kept at 23–25°C in a recirculation system with filtered water disinfected by ultraviolet light, fed with pelleted dry food (Hikari, Kyorin CO., LTD., Japan) daily and acclimated to this environment for at least 3 weeks prior to use for all experiments except **Figures 2–4**. Carp were also bred and reared in the Aquatic Research Facility of WU, the Netherlands. Here, carp were raised at 23°C in recirculating UV-treated tap water, fed pelleted dry food daily (Skretting, Nutreco) and utilized for experiments in **Figures 2–4**. Since G-CSF paralogs of Asian and European common carp show very high sequence identity (98 to 100%), we combined data from NU and WU. Experiments were performed in accordance with the guidelines of NU and WU and with approval of the animal experimental committee of WU.

### Isolation of Carp Tissues and Leukocytes and Purification of Leukocyte Sub-types Such as B Cells, Granulocytes, Macrophages, Thymocytes and Thrombocytes

For tissue and cell isolation, carp were anesthetized with 0.01% Benzocaine (Sigma-Aldrich) or Tricaine Methane Sulfonate (TMS, Crescent Research Chemicals, Phoenix, USA), bled from the caudal vein and euthanized.

Leukocytes were obtained from kidney (head and/or trunk kidney) and spleen. Cell suspensions were obtained by macerating tissues on a sterile mesh in 10 mL of Eagle's minimal essential medium (MEM, Nissui, Tokyo, Japan). Cells were collected by centrifugation at 250 × *g* for 5 min at 4°C, re-suspended in 5 mL of MEM, layered onto a Percoll (1,075 g/cm^3^, GE healthcare) and centrifuged at 430 × *g* for 20 min at 4°C. Cells at the medium/Percoll interface (mononuclear cells) were harvested, washed twice with MEM by centrifugation, re-suspended with E-RDF medium (Kyokuto Pharm. Ind. Co.,Ltd., Tokyo, Japan) containing 20% fetal bovine serum and 2.5% carp serum (E-RDF20/2.5) and passed through 40 μm filter to remove aggregate.

Peripheral blood leukocytes (PBL) were obtained from carp blood. In short, 1 mL of blood was withdrawn from the caudal vein from fish with heparinized syringe, transferred to 9 mL of ice-cold MEM, layered onto a Percoll (1,075) and centrifuged at 430 × *g* for 20 min at 4°C without brakes. Cells at the medium/Percoll interface were harvested, washed twice with MEM by centrifugation and re-suspended with E-RDF20/2.5.

Kidney neutrophils were isolated as described previously (22) with minor modifications. Briefly, trunk kidney cells were layered onto a Percoll discontinuous gradient (1,080 and 1,100 g/cm^3^) and centrifuged at 430 × *g* for 20 min at 4°C. Cells at the 1,080/1,100 interface (neutrophils and erythrocytes) were harvested after complete removal of cells at the upper phase and then washed once. The neutrophil/erythrocyte pellet was treated with 1× red blood cell lysis buffer (150 mM NH_4_Cl, 10 mM KHCO_3_, 0.1 mM EDTA). Cells were washed twice with MEM by centrifugation and re-suspended with appropriate medium for each experiment. The purity of the neutrophils was verified to be >92% by a flow cytometry using a BD FACS Canto (BD Biosciences) and a peroxidase staining according to a DAB oxidization.

Thymocytes (23), macrophages (24), neutrophilic granulocytes (25) were obtained as previously described. Magnetic activated cell sorting (MACS) was used to isolate B cells and thrombocytes from peripheral blood leukocytes (PBLs) using anti-carp IgM [WCI12, (26)] and anti-carp thrombocytes [WCL6, (27)] antibodies and neutrophilic granulocytes from trunk kidney [using monoclonal antibody TCL-BE8; (25)]. The purity of the sorted cells was verified to be >98% by flow cytometry using a BD FACS Canto A (BD Biosciences).

### Identification and *in silico* Analysis of Carp G-CSFs

Genomic loci of carp G-CSF were predicted by the Augustus gene prediction server using information on genes (*med24, psmd3*, and *kpnb1*) known to be neighboring G-CSF in several other species. Primers were designed against carp genomic sequences encoding putative carp G-CSFs. The complete list of primers used for PCR, RACE PCR, qRT-PCR and recombinant protein expression are listed in Supplementary Tables S2–S4. PCR reactions were performed using cDNA from carp kidney and heart with PrimeSTAR HS DNA polymerase (Takara, Shiga, Japan). Generated amplicons were gel purified using the FastGene Gel/PCR Extraction kit (Nippon genetics, Tokyo, Japan), ligated into the pMD20-T vector (Takara) using the 10 × A-attachment Mix (Toyobo, Osaka, Japan) and the Ligation high ver. 2 (Toyobo) and transformed into the competent *Escherichia coli* DH5α. Positive colonies were identified by colony PCR using the vector specific M13 RV and M4 primers, plasmids isolated using the FastGene Plasmid Mini kit (Nippon genetics) and inserts sequenced using a BigDye terminator v3.1 cycle sequencing kit (Applied Biosystems) and an ABI PRISM 3130 Genetic Analyzer (Applied Biosystems). Sequence analysis was performed using the Genetyx version 11 (Genetic Information Processing Software) and sequences aligned and analyzed using BLAST programs.

G-CSF protein sequences from fish, amphibian and mammals were aligned using Clustal Omega software (EMBL-European Bioinformatics Institute). Signal peptide regions of respective G-CSF proteins were estimated using the SignalP 4.0 server (http://www.cbs.dtu.dk/services/SignalP/) and conserved motifs were predicted using the SMART server (http://smart.embl-heidelberg.de/). Phylogenetic analysis was conducted using the MEGA version 6 software using the neighbor-joining (NJ) method and the Poisson method, and bootstrapped 1,000 times, with values expressed as percentages. The full-length sequences of carp G-CSFa1, G-CSFa2, G-CSFb1, and G-CSFb2 (accession number: MG882495, MG882496, MG882497, and MG882498, respectively) have been submitted to GenBank.

### RT-qPCR Analysis of Gene Expression of Carp G-CSF Paralogs in Healthy Carp Tissues, Different Cell Types and Macrophages Stimulated With Mitogens

To investigate basal gene expression levels of G-CSF paralogs, tissues and leukocytes were collected from healthy carp (detailed in the figure legends), then total RNA was isolated using the RNeasy kit (QIAGEN) including on-column DNase treatment according to the manufacturer's instruction and stored at −80°C. cDNA was synthesized with SuperScript III First Strand Synthesis System (Invitrogen) according to manufacturer's instructions. Real-time quantitative PCR (RT-qPCR) was performed with a Rotor-Gene 6000 (Corbett Research) using ABsolute QPCR SYBR Green Mix (Thermo Scientific). Fluorescence data from RT-qPCR experiments were analyzed using Rotor-Gene software v1.7. The take-off value for each sample and the average reaction efficiencies (*E*) for each primer set were obtained upon Comparative Quantitation Analysis from Rotor Gene Software. The relative expression ratio (*R*) of target genes were calculated based on the average *E* and relative to the s11 protein of the 40 s subunit as a reference gene. Take-off values of samples in which genes of interest were non-detectable were given an arbitrary take-off value of 32.

### Generation of Recombinant Carp G-CSFa1 and G-CSFb1

The portions of the carp G-CSFa1 and G-CSFb1 sequences corresponding to the mature, signal sequence-cleaved peptides were PCR amplified from carp kidney and heart cDNA using primers designed to meet the requirements of the pET-16b expression vector (Novagen). The resulting PCR products were ligated into pMD20-T vector, digested with two restriction enzymes, *Nde*I and *BamH*I, isolated by gel electrophoresis, ligated into the pET-16b which carry an N-terminal 10x His-tag, transformed into competent *E. coli* DH5α. Plasmids containing the in frame insert of carp G-CSFa1 or G-CSFb1 were transformed into the T7 polymerase expressing Rossetta-gami B (DE3) pLysS *E. coli* (Novagen), induced with appropriate IPTG and the optimal induction times and temperatures deduced in pilot runs. The bacteria were scaled up accordingly.

Recombinant carp G-CSFa1 and G-CSFb1 were purified from bacterial cells using Ni-NTA agarose (Qiagen, Hilden, Germany) according to the manufacturer's procedure. Briefly, transformed *E. coli* cells were grown in 100 mL LB medium containing 50 mg/mL ampicillin and 30 mg/mL chloramphenicol to density of OD_600_ = 0.5 at 37°C, and then cooled on ice. Expression of recombinant G-CSFa1 was induced by addition of 0.5 mM IPTG at 37°C for 4 h, and expression of recombinant G-CSFb1 was induced by addition of 0.25 mM IPTG at 25°C for 8 h. After shaking the cultures, cells were harvested, lysed in the lysis buffer (20 mM sodium phosphate, 500 mM NaCl, 50 mM imidazole, pH 7.4, 0.1% Triton-X and protease inhibitor cocktail) and sonicated. The insoluble materials were removed by centrifugation at 9,600 × *g* for 20 min and the supernatants were incubated with 500 μL of Ni-NTA agarose slurry at 4°C for 1–2 h with gentle rotation. The resin was then washed with 30 mL of the wash buffer (20 mM sodium phosphate, 500 mM NaCl, 50 mM imidazole, pH 7.4) on columns. Proteins were eluted from the resin using the elution buffer (20 mM sodium phosphate, 500 mM NaCl, 500 mM imidazole, pH 7.4). Subsequently, recombinant G-CSFa1 and G-CSFb1 were purified with gel-filtration to further clarify and simultaneously to determine their molecular weight under a native condition. Gel-filtration was performed using a Sephacryl S-100 column (HR 16/160, GE Healthcare) and the proteins were eluted with 20 mM sodium phosphate buffer containing 300 mM NaCl, pH 7.4 in 0.5 mL/min flow rate. The purified proteins were then passed through Pierce High Capacity Endotoxin Removal Columns (Thermo Fisher Scientific) to remove potential traces of endotoxin, buffer exchanged to 1x PBS with 0.05% BSA, filter sterilized (0.22 μm) and stored at 4°C or −80°C until use. Residual endotoxin was checked to be less than 5 pg/mL according to a Limulus ES-II Single Test (Wako, Osaka, Japan). A Bradford assay (Thermo Fisher Scientific) was performed according to manufactures' directions to determine protein concentration.

Recombinant carp erythropoietin (EPO), kit ligand A (KITLA) and thrombopoietin (TPO) were produced and purified as described previously (28, 29).

### Cell Proliferation Assay

Freshly isolated carp head and trunk kidney leukocytes from 4 individuals were adjusted to a concentration of 4–8 × 10^5^ cells/mL in E-RDF20/2.5 medium. Fifty microlitre of this cell suspension was added to each well of a 96-well plate to which 50 μL of treatment in E-RDF20/2.5 medium was added. Treatments consisted of the E-RDF medium (negative control), 25% cell conditioned medium (CCM) derived from the carp kidney leukocyte culture in which macrophages develop [positive control, (30)], a combination of 100 ng/mL TPO and 100 ng/mL KITLA (positive control), recombinant G-CSFa1 or G-CSFb1 at final concentrations of 500, 100, 20, 4, 0.8, 0.16 ng/mL and heat-inactivated (98°C for 30 min) recombinant G-CSFa1 and G-CSFb1. Cell proliferation was determined using the colorimetric MTT assay (Nacalai Tesque, Kyoto, Japan) which was first shown to provide comparable data for different leukocyte cell types (data not shown). Briefly, 10 μL of MTT reagent was added to each well and plates were incubated at 30°C for 5 h to develop a coloration reaction depend on live cell number. One hundred microlitre of solubilization solution (acid-isopropanol) was then added to each well and plates were sealed and kept at 30°C for 12 h. Cell proliferation was determined on days 0, 3, 6, and 9, and plates were read at absorbance of 570 nm and 650 nm as a reference using Multiskan GO microplate reader (Thermo Fisher Scientific). Values obtained at absorbance of 650 nm from each well were subtracted from values obtained at absorbance of 570 nm from each well.

### RT-qPCR Analysis of Gene Expressions in Cells Treated With Recombinant G-CSFa1 and/or G-CSFb1

Freshly isolated carp kidney leukocytes were seeded into 24-well plates in 0.5 mL of E-RDF20/2.5 at a concentration of 4 × 10^5^ cells/mL. Cells were either treated with the medium (untreated control), recombinant G-CSFa1 (100 ng/mL final concentration), recombinant G-CSFb1 (100 ng/mL final concentration) or the combination of 100 ng/mL G-CSFa1 and 100 ng/mL G-CSFb1 in the E-RDF20/2.5 for 12 h and 4 days at 30°C. Following incubation, cells were collected, total RNA was isolated using the NucleoSpin RNA kit (Macherey-Nagel, Düren, Germany) and reverse transcribed into cDNA using the Omniscript RT kit (Qiagen) according to manufacturer's instructions. Quantitative PCR was performed for carp transcription factors known to be involved in early hematopoiesis (*gata2*) (31), myelopoiesis (*pu.1, cebpa* and *irf8*) (32–35), erythropoiesis (*gata1*) (36) and lymphopoiesis (*gata3* and *pax5*) (37, 38) and myeloid cell markers (*gcsfr1, gcsfr2, csf1r* and *mpx*) (22, 35) using a Thermal Cycler Dice Real Time System *II* (Takara). Beta-actin (β*-actin*) was employed as an endogenous control. Quantitative PCR cycling conditions were 95°C for 30 s followed by 40 cycles of 95°C for 5 s and 60°C for 30 s. Data were analyzed using the Thermal Cycler Dice Real Time System software (Takara) and is represented as the average of the four fish (*n* = 4) with standard deviation.

Likewise, kidney neutrophils from carp were treated with the medium, G-CSFa1 and G-CSFb1 in the E-RDF20/2.5 for 6 h at 30°C, RNA isolated, and cDNA synthesis as described above. Q-PCR was performed for carp chemokine receptors (*cxcr1, cxcr2* and *cxcr4*) (39, 40) and NADPH oxidase components (*gp91*^*phox*^, *p22*^*phox*^, *p40*^*phox*^, *p67*^*phox*^, and *p47*^*phox*^) (41). Beta-actin (β*-actin*) was employed as an endogenous control. Quantitative PCR cycling conditions were 95°C for 30 s followed by 40 cycles of 95°C for 5 s and 60°C for 30 s. Data were analyzed using the Thermal Cycler Dice Real Time System software (Takara) and is represented as the average of the three fish (*n* = 3) with standard deviation.

### Semi-solid Colony-Forming Unit Assay

Freshly isolated leukocytes from carp kidney, spleen or peripheral blood were re-suspended to 2 × 10^5^ cells/mL in E-RDF medium containing 40% FBS and 5% carp serum. Colony-forming unit assay using semi-solid media was carried out as previously described (28) with minor modifications. Briefly, a complete methylcellulose medium was prepared by mixing a 2.0% methylcellulose stock solution with an equal amount of the cell suspension. In some cases (for experiments of colony counting based on the morphology), a complete 0.45% agarose medium was prepared by mixing an 1.8% agarose solution; 2 × E-RDF medium; and the cell suspension in the volume ratio of 1:1:2. Then, 2.4 mL of the cell suspension/complete semi-solid medium was added to a 5 mL tube with a 2.5 mL syringe and 16-gauge blunt-end needle, along with cytokines or PBS. Tubes were tightly capped and the solution gently mixed. One milliliter of the solution (in duplicate) was aliquoted onto a solid E-RDF medium containing 0.45% agarose (Lonza), 20% FBS and 2.5% carp serum in a 35 mm dish or a 6-well plate. Dishes and Plates were incubated at 30°C with an additional 5% CO_2_ atmosphere and 100% humidity for 7–13 days, followed by examination for colony formation.

The number of progenitor cells in each organ was estimated according to the formula described below.

$$\begin{array}{l} No.\;of\;progenitors\;=\;\left(Total\;No.\;of\;leukocytes\;from\;each\;organ\right)\\ \times \;\left(No.\;of\;colonies\;forming\;per\;plate\right)/(No.\;of\;leukocytes\;plated). \end{array}$$

### Characterization of Colony Cells

Cell colonies formed in the methylcellulose media were aspirated by micropipette under a microscope and characterized by morphology, cytochemistry and RT-PCR analyses as previously described (28). In short, the colony cells were re-suspended in MEM, and cyto-centrifuged with a Cytospin (Shandon). Slides were dried, fixed and stained with May-Grünwald Giemsa (MGG, Wako Pure Chemicals, Osaka, Japan) or Peroxidase stain based on the DAB oxidization. For RT-PCR analyses, total RNAs were extracted from each colony cell type using RNeasy Micro Kit (Qiagen) and cDNA was synthesized using Omniscript RT kit (Qiagen). Expression of hematopoietic marker genes was analyzed by PCR using carp specific primers and EmeraldAmp PCR Master Mix (Takara). PCR was conducted as follows: one cycle of 94°C for 1 min, followed by 23 to 38 cycles of denaturation at 98°C for 30 sec, annealing at 58°C for 30 s and elongation at 72°C for 30 s. Colonies treated with recombinant carp erythropoietin (EPO) was utilized for the control group of the erythroid lineage.

### Neutrophil Chemotactic Response to Recombinant Carp G-CSFa1 and G-CSFb1

Neutrophils obtained from carp trunk kidney were washed twice in MEM and adjusted to a final concentration of 1 × 10^6^ cells/mL. The chemotaxis assay was performed using blind well chemotaxis chambers (Neuro Probe, Inc.). Two hundred microliters of different concentrations of recombinant carp G-CSFa1 or G-CSFb1 (1, 10, and 100 ng/mL, final concentrations) in the serum free MEM were added to the bottom well of the chemotaxis chambers, and the bottom chamber was separated from the top chamber using 5 μm pore size polycarbonate membrane filters (Neuro Probe, Inc.). To the top chamber, 200 μL of neutrophil suspension was added. Negative controls consisted of medium alone and the positive control was 10 ng/mL of fMLP (Sigma-Aldrich). The chemokinesis control consisted of 100 ng/mL of G-CSFa1 or 100 ng/mL of G-CSFb1 in both the upper and lower chambers of the chemotaxis apparatus.

The incubation period was 1 h after which the cell suspensions were carefully aspirated from the top chamber and the filters removed and applied bottom side up on a slide glass. Filters were stained with MGG. Chemotactic activity was determined by counting the total number of cells found on the underside of the polycarbonate filters in 20 random fields of view (40 × magnification). Technical duplicates were conducted for all treatments (*n* = 4, biological replicates).

### Respiratory Burst Assay

Respiratory burst assay was performed as previously described (42) with minor modifications. The neutrophils harvested from carp kidney were re-suspended in E-RDF20/2.5 medium at a concentration of 2.5 × 10^6^ cells/mL. Four hundred microliters of the cell suspension was added to each 1.5 mL tubes, and cells were treated or untreated with recombinant carp G-CSFa1 (100 ng/mL) or G-CSFb1 (100 ng/mL) at 25°C for 6 h. Following the incubation, DHR123 (Molecular Probes) was added to the cells at a final concentration of 10 μM and incubated for 5 min to allow the cells to take up the DHR123. PMA (Abcam, Cambridge, UK) was then added at a final concentration of 100 ng/ml. The cells were further incubated for 30 min to allow oxidation of the DHR. All samples were appropriately staggered with respect to timing to accommodate the transient state of oxidized DHR fluorescence. Live cells were gated according to the forward scatter and side scatter parameters. DHR fluorescence was detected in the FITC channel, and the mean values of the FITC fluorescence in neutrophils were normalized to untreated controls.

### *In vivo* Effects of G-CSFa1 and G-CSFb1

Carp were bled 200 μL from the caudal vein using a heparinized syringe 2 days before administration of G-CSF paralogs and blood was centrifuged in a capillary glass tube at 1,500× *g* for 5 min. Leukocytes on top of the erythrocyte layer were obtained, treated with the 1× red blood cell lysis buffer, washed twice with Hanks' balanced salt solution and then used to measure the ratio of neutrophils per total number of leukocytes by flow cytometry analysis, based on forward scatter vs. side scatter parameters. Subsequently, carp were injected intraperitoneal (i.p.) with 100 ng/g body weight of recombinant proteins in 200 μL of 1×PBS, or were injected with 1 × PBS only. After 6, 24 and 48 h, 200 μL of peripheral blood was collected and analyzed as described above. Three fish for each group were examined.

### Statistical Analysis

Raw data of technical replicates were first averaged per individual before statistical analysis was performed. Statistical analysis was performed after log-transformation of datasets that were not normally distributed. Subsequently, normality was assumed and statistical significance was tested using an un-paired Student's *t*-test (independent observations, **Figure 3**) for one-to-one comparisons and a (repeated-measures) one-way analysis of variance (ANOVA) followed by a Dunnet's *post-hoc* test (in **Figures 4, 5A,D,E, 7, 8**). Or, Tukey's HSD (**Figure 2**) and Dunnet's T3 (in case of unequal variances) (**Figure 2**) were used for multiple comparisons. A two-way ANOVA was performed followed by Tukey's *post-hoc* test for multiple comparisons shown in **Figures 9, 10**. Prism 7 software (GraphPad Software, La Jolla, CA, USA) was used. In absence of sphericity, the Geisser-Greenhouse correction was applied. A value of *p* ≤ 0.05 was accepted as significant.

## Results

### Identification of Four Carp G-CSF Paralogs

**Figure 1:**
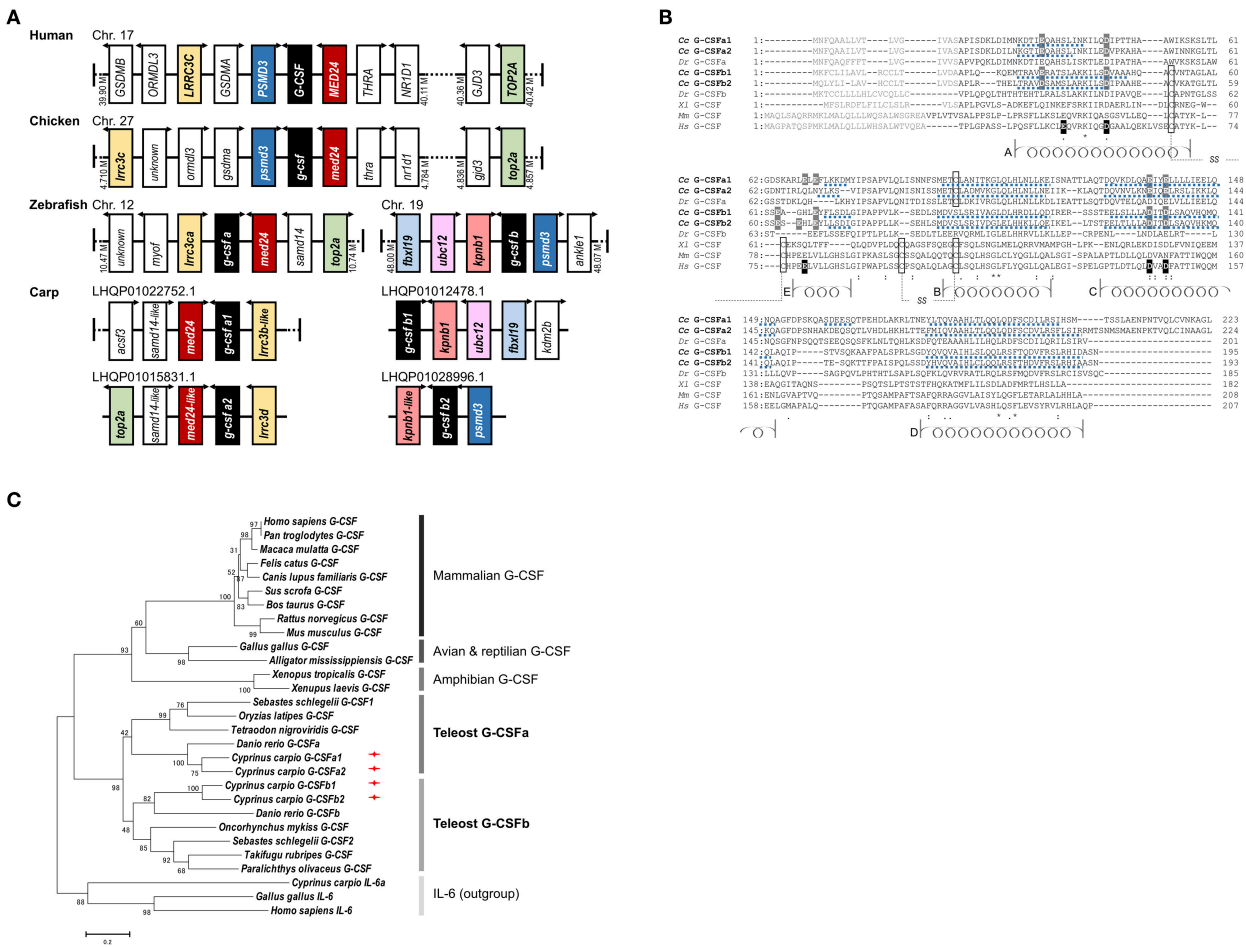
Granulocyte colony-stimulating factor paralogs of common carp. **(A)** Synteny analysis of G-CSF of human, chicken, zebrafish, and carp. The G-CSF locations in human, chicken, and zebrafish are obtained from respective genome resources at NCBI and carp G-CSF locations are obtained from carp genome and transcriptome projects PRJEB7241 and PRJNA73579 at NCBI. **(B)** Alignment of vertebrate granulocyte colony-stimulating factor (G-CSF) amino acid sequences. Complete protein sequences of carp *Cc* G-CSFa1 (*Cyprinus carpio*, MG882495), carp *Cc* G-CSFa2 (MG882496), zebrafish *Dr* G-CSFa (*Danio rerio*, NP_001138714), carp *Cc* G-CSFb1 (MG882497), carp *Cc* G-CSFb2 (MG882498), zebrafish *Dr* G-CSFb (NP_001137226), african clawed frog *Xl* G-CSF (*Xenopus laevis*, Scaffold13265:3008399-3011722), mouse *Mm* G-CSF (*Mus musculus*, NP_034101), and human *Hs* G-CSF (*Homo sapiens*, NP_000750) were aligned using Clustal Omega. Signal sequences are shown with gray letters and conserved cysteine residues are boxed. In the human G-CSF, helices (A to E) are denoted with coils, and Glutamic acid (E) and Aspartic acid (D) residues representing major interfaces with G-CSF receptor are highlighted in black. In the carp G-CSFs, predicted alpha helical regions are underlined with a broken line, modeled on the structure of human G-CSF, and residues expected to interact with the receptor are highlighted in gray. Amino acids that are conserved in all sequences are denoted with an asterisk (^*^), strong similarity with a colon (:), and weak similarity with a period (.). **(C)** Phylogenetic analysis of mammalian, avian, reptilian, amphibian and teleost fish G-CSF proteins. Phylogenetic analysis included carp G-CSFa1 (*C. carpio*, MG882495); carp G-CSFa2 (MG882496); carp G-CSFb1 (MG882497); carp G-CSFb2 (MG882498); zebrafish G-CSFa (*D. rerio*, NP_001138714); zebrafish G-CSFb (NP_001137226); rockfish G-CSF1 (*S. schlegelii*, BAH56611); rockfish G-CSF2 (BAH56612); medaka G-CSF (*O. latipes*, XP_004080425); green-spotted puffer G-CSF (*T. nigroviridis*, CAG04394); rainbow trout G-CSF (*O. mykiss*, CAQ42965); fugu G-CSF (*T. rubripes*, XP_003965085); flounder G-CSF (*P. olivaceus*, BAE16320); african clawed frog G-CSF (*X. laevis*, Scaffold13265:3008399-3011722); tropical clawed frog G-CSF (*X. tropicalis*, XP_002940261); alligator G-CSF (*A. mississippiensis*, XP_006270858); chicken G-CSF (*G. gallus*, NP_990610); human G-CSF (*H. sapiens*, NP_000750); chimpanzee G-CSF (*P. troglodytes*, XP_009430519); rhesus monkey G-CSF (*M. mulatta*, XP_001095097); cat G-CSF (*F. catus*, NP_001009227); dog G-CSF (*C. lupus familiaris*, XP_005624600); pig G-CSF (*S. scrofa*, XP_005653977); cattle G-CSF (*B. taurus*, NP_776453); rat G-CSF (*R. norvegicus*, NP_058800); mouse G-CSF (*M. musculus*, NP_034101); and outgroup including carp interleukin-6a (IL-6a, AGR82313); chicken IL-6 (NP_989959); and human IL-6 (NP_000591). Phylogenetic analysis was conducted using the neighbor joining method in MEGA version 6 and bootstrapped 1,000 times with bootstrap values expressed as percentages.

The presence of four G-CSF paralogs in the genome of common carp was expected as common carp has undergone a WGD event compared to the zebrafish (21), in which two G-CSF paralogs are already present. Referring to carp genome and transcriptome databases (project no. PRJEB7241 and PRJNA73579) at the National Center for Biotechnology Information (NCBI), four putative loci encoding carp G-CSF homologs were found next to conserved genes *PSMD3, MED24*, or *LRRC3* (Figure 1A). Synteny of each paralog is highly conserved with either the zebrafish G-CSFa locus on chromosome 12 or the GCSFb locus on chromosome 19.

The complete open reading frames (672, 675, 588, and 582 bp) of four carp paralogs' cDNA transcripts, respectively, encoding 224, 225, 196, and 194 amino acids with 5 exons were obtained (Figure 1B and Supplementary Figures S1A–D). Despite quite low sequence identity and similarity (Supplementary Table S1), carp G-CSF paralogs share a similar predicted structure and one of the copies (G-CSFa1) was predicted to have an additional helical region from Ser^160^ to Ser^164^ which is acidic amino acid residue (Asp and Glu)-abundant (Figure 1B and Supplementary Figure S2). Carp G-CSF paralogs all possess the consensus domain of Pfam IL6/GCSF/MGF protein family, whereas the four cysteine residues involved in two disulfide bonds are not conserved. Carp G-CSF copies also share conserved acidic amino acids involved in major ligand-receptor binding demonstrated in human G-CSF, while there is no acidic amino acid residue near the α-helix E in carp G-CSFa2 (Figure 1B). Phylogenetic analysis revealed that all the G-CSFs were found to form a single evolutionary clade outside a related cytokine interleukin-6, suggesting that the G-CSFs are orthologous. Taking into account there may be G-CSF paralogs present in the teleost species shown that have not yet been reported, each of the four carp paralogs did cluster with either teleost G-CSFa or G-CSFb paralogs (Figure 1C). Hence, based on clustering and the conserved synteny, we named the four carp G-CSF paralogs as G-CSFa1, G-CSFa2, and G-CSFb1, G-CSFb2.

### Carp *g-csf* Paralogs Show Differential Expression in Immune Tissues and Cells

**Figure 2:**
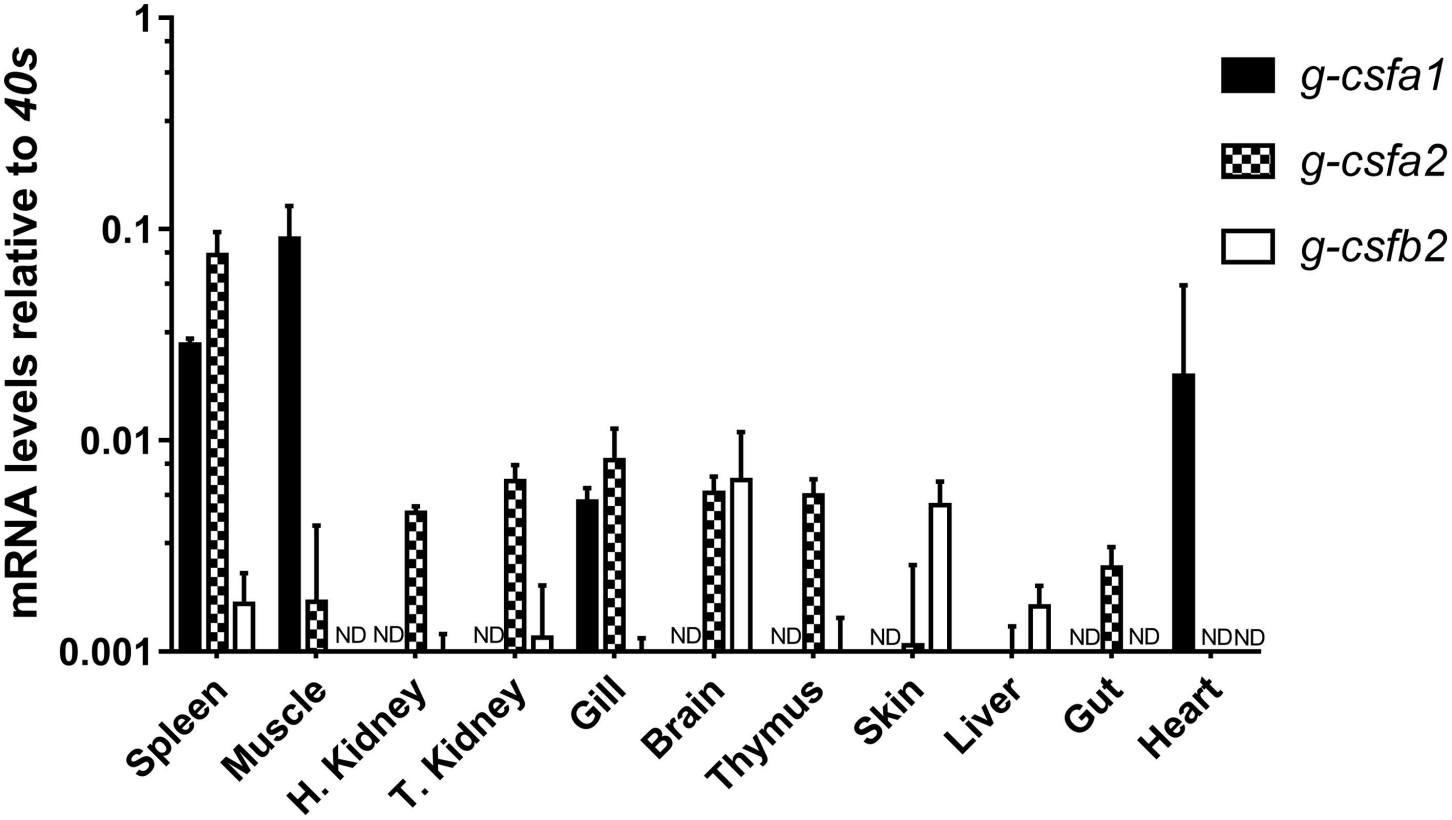
Quantitative mRNA expression analysis of carp *g-csf* paralogs in carp tissues. Basal gene expression of carp G-CSF paralogs in spleen, muscle, head kidney, trunk kidney, gill, brain, thymus, skin, liver, gut, and heart. Basal expression levels were determined relative to the s11 protein of the 40s subunit (*40s*) as a reference gene and are presented as mean + standard deviation (*n* = 3, except thymus, *n* =2). *G-csfb1* expression was non-detectable in all tissues examined (ND indicates “non-detectable”). Significant differences in expression between tissues were determined using one-way ANOVA followed by Tukey's HSD (*g-csfa1* and *g-csfb2*) or Dunnet's T3 *post-hoc* test for unequal variances (*g-csfa2*).

Assessment of basal *g-csfa1* expression in tissues from healthy carp revealed generally very low expression of *g-csfa1* in most tissues, with significantly higher gene expression in spleen, muscle and gill (Figure 2). *G-csfa2* was significantly higher expressed in spleen than in gill, brain, thymus, trunk-kidney and head-kidney (Figure 2). Basal expression levels of *g-csfb1* and *g-csfb2* were generally low or non-detectable in most carp tissues examined (Figure 2).

**Figure 3:**
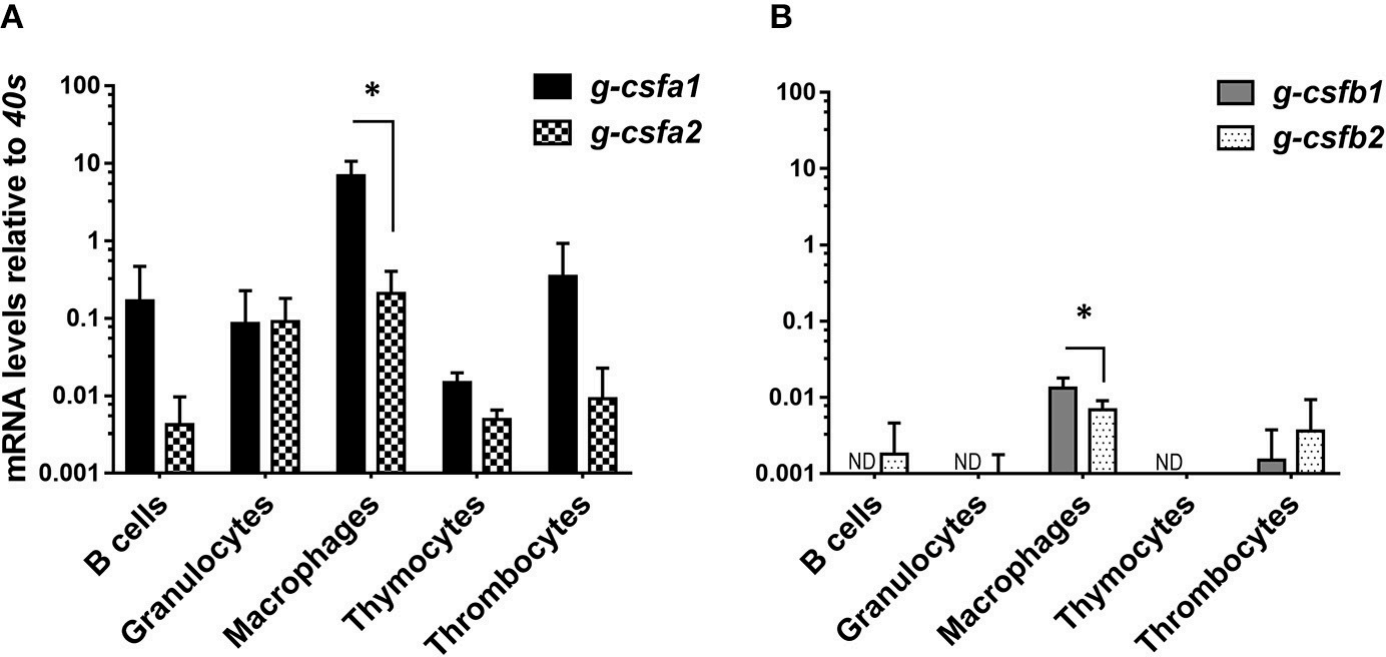
Quantitative mRNA expression analysis of carp *g-csf* paralogs in immune cells. Basal gene expression of carp *g-csfa1* and *g-csfa2* **(A)** and *g-csfb1* and *g-csfb2* **(B)** in sorted cells from healthy carp. Basal expression levels were determined relative to the s11 protein of the 40s subunit (*40s*) as a reference gene and presented as mean + standard deviation (*n* = 4 except thymocytes and thrombocytes *n* = 2). Statistical significance within the macrophage group was determined using an un-paired Student's *t*-test. Asterisks (^*^) denotes significance (*p* < 0.05) between indicated genes. ND indicates “non-detectable”.

At basal levels, g-csfa expression is markedly higher than g-csfb expression in all immune cells examined. Strikingly, all *g-csf* paralogs were highest expressed at basal level in macrophages, indicating these cells as the major producers of G-CSF, comparable to mammalian macrophages. Within macrophages, g-csfa1 and g-csfb1 were significantly higher expressed compared to their respective counterparts (Figures 3A,B). Remarkably, a clear expression of *g-csfa1* was observed also in thrombocytes (Figure 3A).

### Expression of Carp *g-csf* Paralogs Are Immediately Enhanced After Stimulation

**Figure 4:**
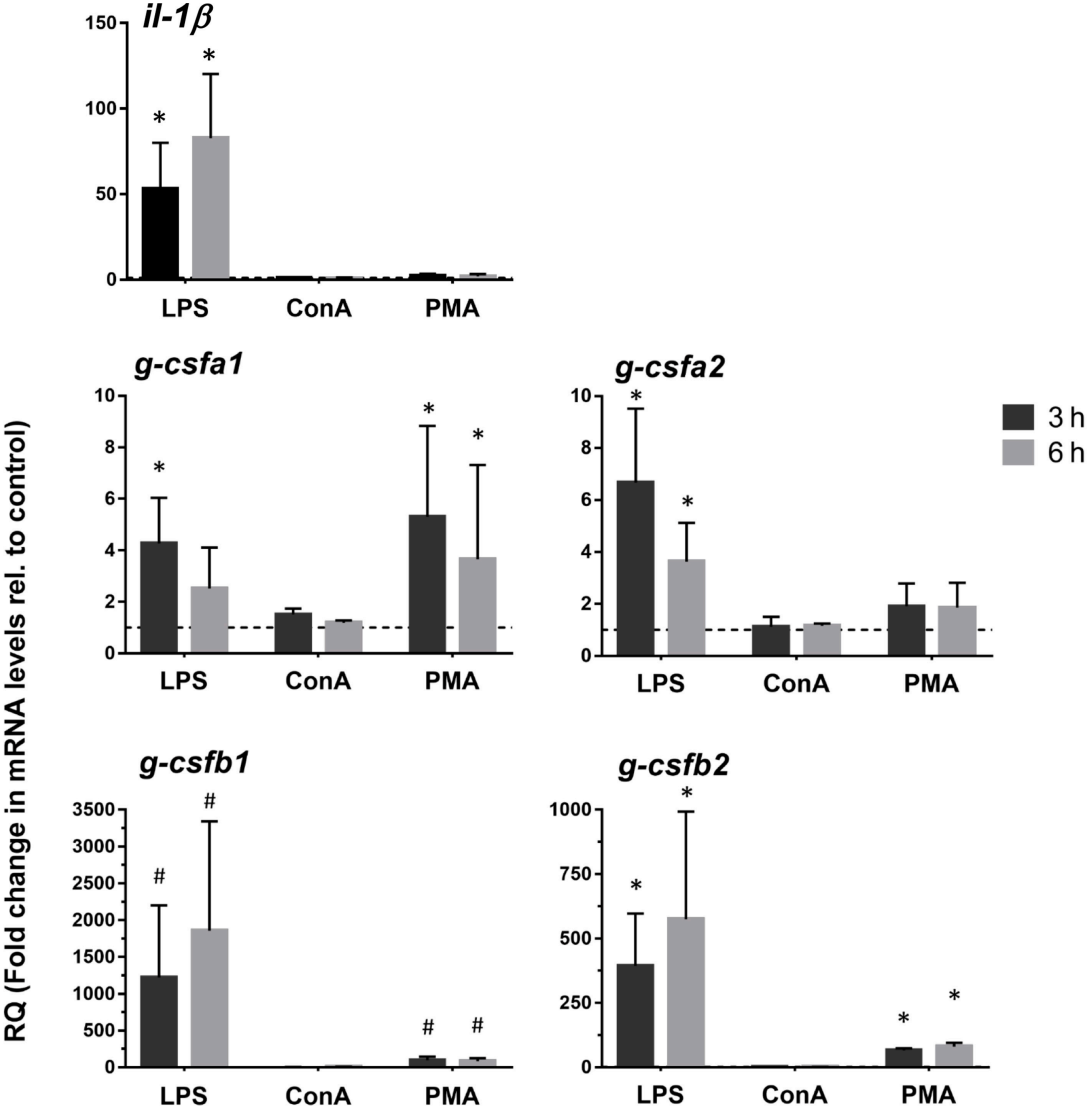
Quantitative expression analysis of carp *g-csf* paralogs in stimulated macrophages. Gene expression analysis of head kidney-derived macrophages stimulated for 3 or 6 h with 50 μg/mL lipopolysaccharide (LPS), 10 μg/mL Concanavalin A (ConA) or 1 μg/mL phorbol myristate acetate (PMA). Gene expression was normalized relative to the *s11* protein of the *40s* subunit as a reference gene and expressed relative to unstimulated timepoint controls (dashed line at *y* = 1). Data are presented as mean + standard deviation (*n* = 4). Significant differences compared to unstimulated timepoint controls were determined using one-way ANOVA followed by Dunnet's *post-hoc* test, (*p* < 0.05) are denoted by asterisks (^*^). Non-detectable samples were given an arbitrary value of CT = 32. Hash mark (#) indicate significant differences using these arbitrary values.

In order to determine induction of the different G-CSF paralogs upon antigenic stimulation, we investigated expression levels in freshly isolated kidney leukocytes and head kidney-derived macrophages following the stimulation with LPS, ConA, PMA and poly I:C (only freshly isolated kidney leukocytes). In freshly isolated kidney leukocytes, all paralogs were highly up-regulated after stimulation with LPS and the combination of ConA/PMA at 3 and 6 h but not after stimulation with Poly I:C (Supplementary Figure S3). Likewise, in the cultured macrophages gene expressions of the four paralogs were clearly enhanced by LPS and PMA stimulations (Figure 4). Despite non-detectable *g-csfb1* transcripts, its gene expression was induced with LPS stimulation. Interestingly, in macrophages both *g-csfa1* and *g-csfa2* are relatively high expressed at basal level (Figure 3) and appear to show a relatively small increase upon stimulation with LPS (Figure 4), whereas *g-csfb1* and *g-csfb2*, which are relatively low or non-detectable at basal level (Figure 3), show a large increase in gene expression after LPS stimulation (Figure 4). We could also show that expression in macrophages of interleukin-1 beta, which is a pro-inflammatory cytokine, was significantly up-regulated after LPS stimulation (Figure 4).

### Recombinant Carp G-CSFa1 and G-CSFb1 Are Monomeric Forms

Based on expression levels in macrophages and the clear induction in stimulated macrophages, we chose to express two copies, G-CSFa1 and G-CSFb1, to investigate their function. Recombinant G-CSFa1 and G-CSFb1 purified using Ni-affinity chromatography were passed through a gel filtration column under a non-denaturing condition to calculate their molecular weights. As a result, the molecular weights of G-CSFa1 and G-CSFb1 were estimated to 25,275 and 22,355, similar to the deduced values based on their primary structures and similar to the result of SDS-PAGE under the denaturing condition, indicating that both recombinants form monomers (Supplementary Figure S4).

### Both G-CSFa1 and G-CSFb1 Induce Proliferation of Kidney Leukocytes, but Only G-CSFb1 Induce Differentiation of Cells Along the Neutrophil Lineage

**Figure 5:**
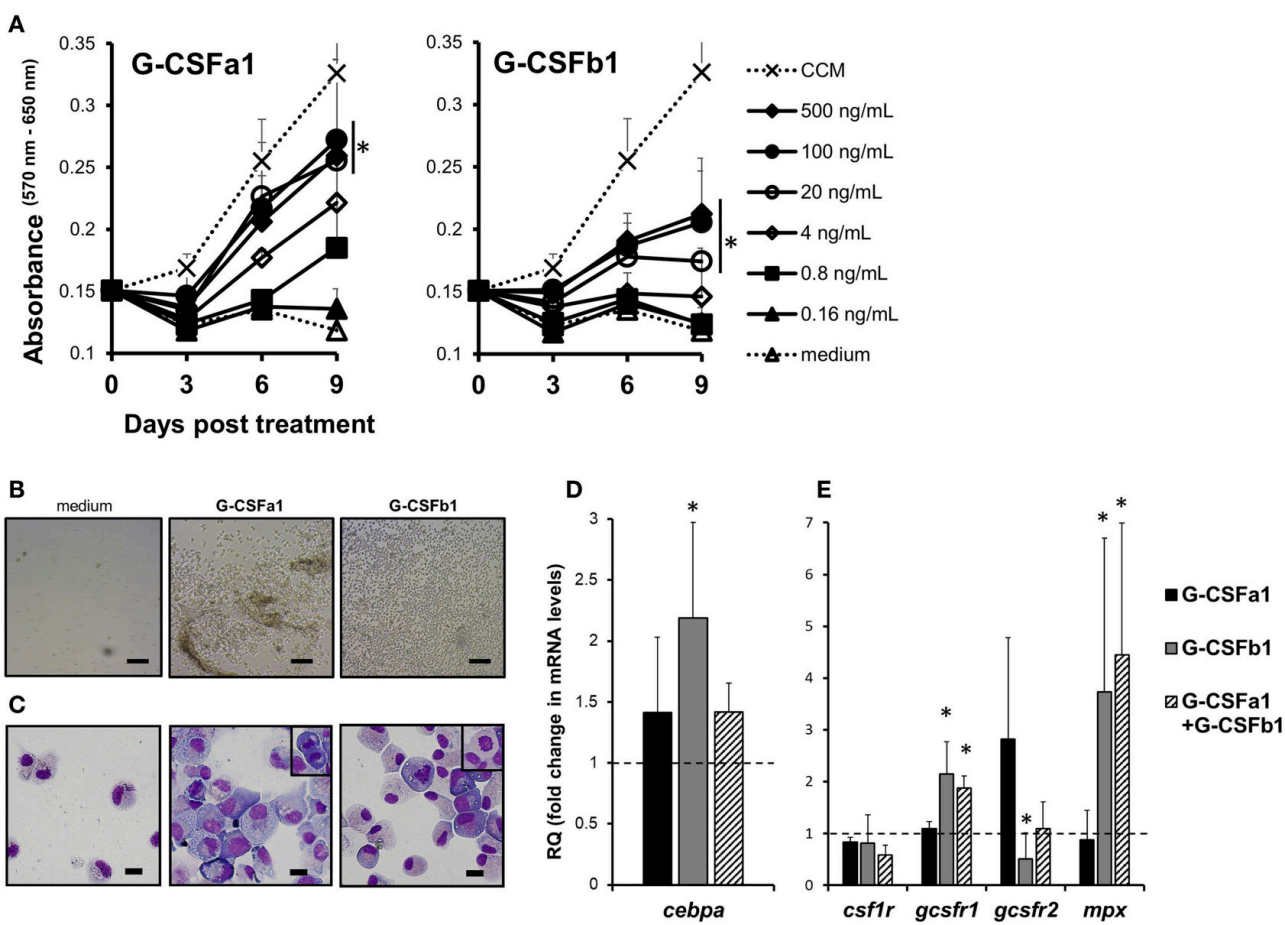
Proliferation and differentiation of carp kidney neutrophilic granulocyte-like cells. **(A)** Proliferative response of carp kidney leukocytes (20,000 cells) treated with medium alone, cell conditioned medium (CCM) derived from kidney leukocyte cultures in which macrophages develop, or recombinant carp G-CSF paralogs at different doses. Live cells treated with each stimulus were measured with the MTT assay at day 0, 3, 6, and 9 in the culture. Absorbance values at 650 nm were subtracted from experimental absorbance values at 570 nm in each well. Each point in the graphs represents mean + standard deviation (*n* = 4). Significant differences compared to medium-treated controls in day 9 were determined using one-way ANOVA followed by Dunnet's *post-hoc* test, (*p* < 0.05) are denoted by asterisks (^*^). **(B)** Photomicrographs of liquid cultures in the absence (medium only) or presence of recombinant G-CSFa1 and G-CSFb1 after 8 days of culture. Scale bars indicate 100 μm. **(C)** May-Grunwald Giemsa staining of kidney cells after 8 days culture in the absence or presence of G-CSFa1 and G-CSFb1. Mitotic figures were frequently observed (small enclosure). Scale bars indicate 10 μm. **(D)** Quantitative gene expression analysis of carp transcription factors involved in granulopoiesis (*cebp*α) in carp kidney leukocytes treated or untreated with G-CSFa1 (100 ng/mL), G-CSFb1 (100 ng/mL) or a combination of G-CSFa1 (100 ng/mL) and G-CSFb1 (100 ng/mL) for 12 h. The mRNA levels were calculated using β*-actin* as a reference gene. Data were normalized to the control cells (dashed like at y = 1) and mean + standard deviation is shown (*n* = 4). Significant differences compared to unstimulated controls were determined using one-way ANOVA followed by Dunnet's *post-hoc* test, (*p* < 0.05) are denoted by asterisks (^*^). **(E)** Quantitative gene expression analysis of myeloid cytokine receptors and myeloperoxidase in carp kidney leukocytes treated or untreated with G-CSFa1, G-CSFb1 or a combination of G-CSFa1 and G-CSFb1 for 4 days. The mRNA levels were calculated using β*-actin* as a reference gene. Data were normalized to the control cells (dashed line at *y* = 1) and mean + standard deviation is shown (*n* = 4). Significant differences compared to unstimulated controls were determined using one-way ANOVA followed by Dunnet's *post-hoc* test, (*p* < 0.05) are denoted by asterisks (^*^).

Carp kidney leukocytes treated with the cell conditioned medium containing macrophage growth factor(s) and recombinant TPO plus KITLA exhibited active proliferation, indicating that there are heterogeneous hematopoietic progenitors in the kidney leukocytes (Figure 5A and Supplementary Figure S5). Treatment of carp kidney leukocytes with 0.8, 4, 20, 100, and 500 ng/mL of G-CSFa1 induced a dose-dependent proliferative response, with the highest proliferation observed in cells treated with more than 20 ng/mL of G-CSFa1, whereas heat-inactivated (98°C for 30 min) G-CSFa1 had no effect. Likewise, treatment of kidney leukocytes with 4, 20, 100, and 500 ng/mL of G-CSFb1 induced a dose-dependent proliferative response, with the highest proliferation observed in cells treated with more than 100 ng/mL of G-CSFb1, whereas heat-inactivated G-CSFb1 had no effect (Figure 5A and Supplementary Figure S5). Furthermore, treatment of kidney leukocytes with a combination of 100 ng/mL of G-CSFa1 and 100 ng/mL of G-CSFb1 enhanced the proliferative response compared with those cells treated with G-CSFa1 alone or G-CSFb1 alone (data not shown).

A lot of growing cells treated with G-CSFa1 adhered onto the plastic and with each other, whereas cells treated with G-CSFb1 exhibited low adhesive property and dispersed (Figure 5B). Morphologically, most cells treated with G-CSFa1 for 8 days were blast-like cells, having a basophilic cytoplasm and round to oval nuclei (Figure 5C). In contrast, the cells treated with G-CSFb1 for 8 days appeared to be at different developmental stages from myeloblast-like to metamyelocyte-like (Figure 5C). Most growing cells with each treatment were ascertained to be myeloid cells by staining with TCL-BE8 monoclonal antibody which mainly binds to carp neutrophils (43) (data not shown).

To characterize the roles of G-CSFa1 and G-CSFb1, we examined the gene expressions of transcription factors (TFs) and cell surface markers involved in the development of various cell lineages in carp kidney leukocytes treated with G-CSFa1, G-CSFb1 and a combination of G-CSFa1 and G-CSFb1. The mRNA levels of the TFs involved in myelopoiesis (*pu.1, cebp*α and *irf8*), early hematopoiesis (*gata2*), erythropoiesis (*gata1*) and lymphopoiesis (*gata3* and *pax5*) in cells treated with or without G-CSFa1, G-CSFb1 and a combination of them for 12 h were analyzed by quantitative PCR. Kidney cells treated with G-CSFa1 did not undergo any change of TFs mRNA levels. On the other hand, kidney cells treated with G-CSFb1 exhibited a significant up-regulation of *cebp*α mRNA levels compared to those of the medium-treated controls (Figure 5D), while other TFs tested showed no significant change (data not shown). Cells treated with the combination of G-CSFa1 and G-CSFb1 also showed a moderate up-regulation of *cebp*α levels compared to those of the controls (Figure 5D). Next, we examined whether G-CSFa1 and G-CSFb1 modulate expression of myeloid cytokine receptors and neutrophil-specific myeloperoxidase in carp kidney cells. The mRNA levels of *csf1r, gcsfr1, gcsfr2*, and *mpx* in cells treated with the same treatments for 4 days were analyzed by quantitative PCR. Expression of *csf1r*, which is the macrophage colony-stimulating factor receptor gene, in the kidney cells was unaffected with any treatment examined (Figure 5E). *Gcsfr1* and *mpx* expression in the kidney leukocytes was up-regulated with the treatment of G-CSFb1 alone and the combination of G-CSFa1 and G-CSFb1, but not with G-CSFa1. On the other hand, *gcsfr2* expression in the kidney leukocytes shows a trend toward upregulation with G-CSFa1 treatment but downregulation with G-CSFb1 treatment (Figure 5E).

### G-CSFb1 Stimulates Granulocyte Colony Formation and Cooperates With G-CSFa1 to Stimulate Granulocyte/Macrophage Colony Formation

**Figure 6:**
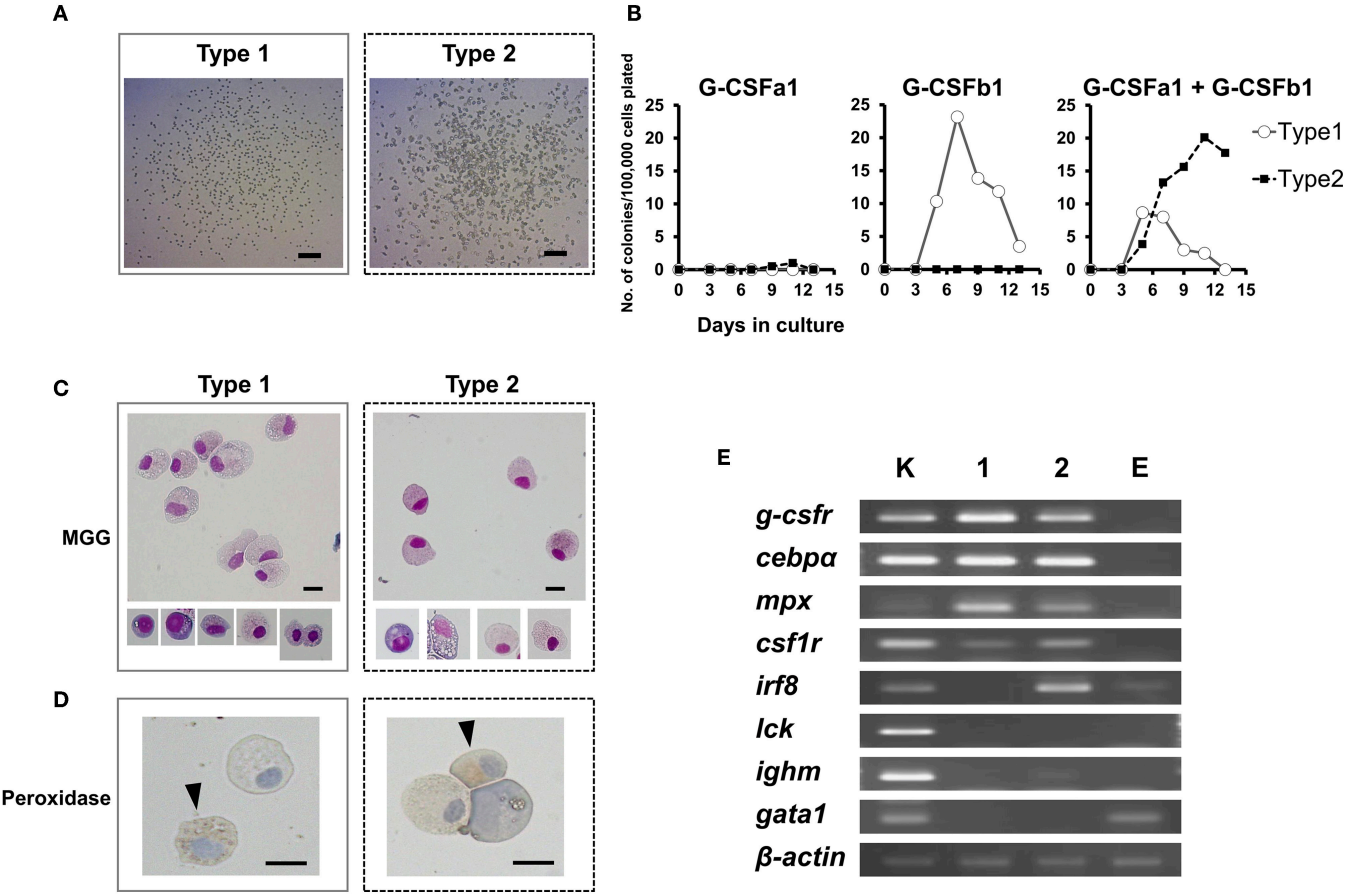
Colony formation of kidney cells in response to recombinant carp G-CSFa1 and G-CSFb1. **(A)** Colony-formation of kidney cells in response to G-CSF paralogs. Overall two types of colonies (type 1 and type 2) were observed. Bars indicate 200 μm. **(B)** Colony counts during semi-solid culture of kidney cells (1 × 10^5^) in the presence of 100 ng/mL G-CSFa1 alone, 100 ng/mL G-CSFb1 alone, or a combination of 100 ng/mL G-CSFa1 and 100 ng/mL G-CSFb1. Each point indicates mean colony counts from 4 individual fish under each condition. Cultures scored every 2 days between 3 and 13 days of incubation. **(C)** May-Grünwald Giemsa (MGG) staining of colony cells (type 1; left and type 2; right). Bars indicate 10 μm. **(D)** Peroxidase-staining of cells obtained from type 1 colonies (left) and type 2 colonies (right), counterstained with Mayer's Hematoxylin. Arrow heads indicate myeloperoxidase-positive cells. Bars indicate 10 μm. **(E)** RT-PCR analysis for expression of lineage-associated marker genes in type 1 (lane 1) and type 2 (lane 2) colony cells. cDNA from carp kidney leukocytes was used as a positive control (lane K). cDNA from cells cultured in the presence of 100 ng/mL carp EPO was utilized for the control group of the erythroid lineage (lane E).

In order to further examine the hematopoietic function G-CSFa1 and G-CSFb1 and identify granulocyte progenitor cells, we used an *in vitro* methylcellulose/agarose colony assay system. As expected, plating of carp kidney leukocytes (100,000 cells) without addition of cytokine resulted in no colony formation (data not shown). In the presence of G-CSF paralogs, overall two types of colonies appeared (Figure 6A). Surprisingly, when carp kidney leukocytes were cultured with G-CSFa1 alone, few colony formations were observed at any dose (Figure 6B left). On the other hand, in the presence of G-CSFb1, approximately 25 homogeneous colonies were formed after 7 days of the incubation (Figure 6B middle). These colonies consisted of uniform small round cells scattered (type 1, Figure 6A left). When kidney cells were cultured with a combination of G-CSFa1 and G-CSFb1, morphologically two kinds of colonies were observed. One appeared to be similar to the type 1 colonies formed in the presence of G-CSFb1 alone, the other seemed to consist of roughly agminated cells with distinct sizes and shapes (type 2, Figure 6A right). Approximately ten type 1 colonies per 100,000 cells plated were formed at day 5 to 7 in the culture and then gradually disappeared. The peak of type 2 colony formation (about 20 colonies per 100,000 cells plated) was observed after 11 days of cultivation (Figure 6B right). Both type 1 and type 2 colony cells consisted of morphologically neutrophil lineage cells at distinct developmental stages, which are myeloperoxidase-positive and –negative (Figures 6C,D). To characterize colony types, the expression of lineage-associated marker genes was analyzed. Figure 6E shows a typical expression patterns in type 1 and type 2 colonies. Type 1 colonies treated with G-CSFb1 alone or G-CSFa1 plus G-CSFb1 highly expressed *g-csfr, cebp*α, and *mpx* mRNAs involved in neutrophil development and slightly expressed *csf1r* which is the macrophage colony-stimulating factor receptor gene, but did not express other genes examined, indicating that type 1 colonies are derived from the progenitor cells corresponding to mammalian granulocyte colony-forming units (CFU-G). Type 2 colonies treated with the combination of G-CSFa1 and G-CSFb1 highly transcribed not only neutrophil-specific marker genes but also monocyte/macrophage lineage markers *csf1r* and *irf8*, suggesting that type 2 colonies are derived from the progenitors corresponding to mammalian granulocyte/macrophage CFU (CFU-GM) (Figure 6E).

### Granulocyte/Macrophage Progenitors and Granulocyte Progenitors Are Localized in the Head Kidney and Trunk Kidney but Not in the Spleen of Carp

To assess the contribution of hematopoietic organs to the neutrophil development in common carp, a myeloid colony forming assay was performed. Leukocytes were harvested from head kidney, trunk kidney, spleen and peripheral blood of adult carp (10 to 15 cm in length). Approximately 1 × 10^5^ cells were cultured in the methylcellulose/agarose media in the presence or absence of 100 ng/mL G-CSFa1 plus 100 ng/mL G-CSFb1 and colony counts were performed after 6–11 days in the culture. PBLs and splenocytes did not form any colonies regardless of addition of cytokine or not. Conversely, cells from head kidney and trunk kidney formed about 25 to 40 colonies of each of type 1 and type 2 in the presence of both G-CSFa1 and G-CSFb1. Total number of CFU-G and CFU-GM in each organ was estimated as Table 1.

**Table 1:** The number of type 1 and type 2 colonies formed from 100,000 cells in head kidney, trunk kidney, spleen, and PBLs in the semi-solid culture with the combination of 100 ng/mL G-CSFa1 and 100 ng/mL G-CSFb1.

	**Head kidney**		**Trunk kidney**		**Spleen**		**PBLs**	
	**Type 1 (CFU-G)**	**Type 2 (CFU-GM)**	**Type 1 (CFU-G)**	**Type 2 (CFU-GM)**	**Type 1 (CFU-G)**	**Type 2 (CFU-GM)**	**Type 1 (CFU-G)**	**Type 2 (CFU-GM)**
Mean of colonies formed in the presence of both G-CSFa1 and G-CSFb1 (± SEM)	29.1 ± 4.2	34.5 ± 4.5	22.9 ± 3.5	34.0 ± 3.8	0	0	0	0
Mean of leukocytes in each whole organ	9.24 × 10^6^		8.94 × 10^6^		3.60 × 10^5^		ND	
No. of progenitors estimated (± SEM)	2,689 ± 388	3,188 ± 416	2,047 ± 313	3,039 ± 340	0	0	0	0

*Data were obtained from duplicate cultures in the presence or absence of both 100 ng/mL G-CSFa1 and 100 ng/mL G-CSFb1 and shown only from the culture with the cytokines (n = 4). Type 1 colonies were counted at 7 days of cultivation. Type 2 colonies were counted at 10 days of cultivation. ND, not determined*.

### G-CSFa1 and G-CSFb1 Directly Induce a Chemotactic Response of Kidney Neutrophils and Up-Regulates the Gene Expression of a Chemokine Receptor *cxcr1*

**Figure 7:**
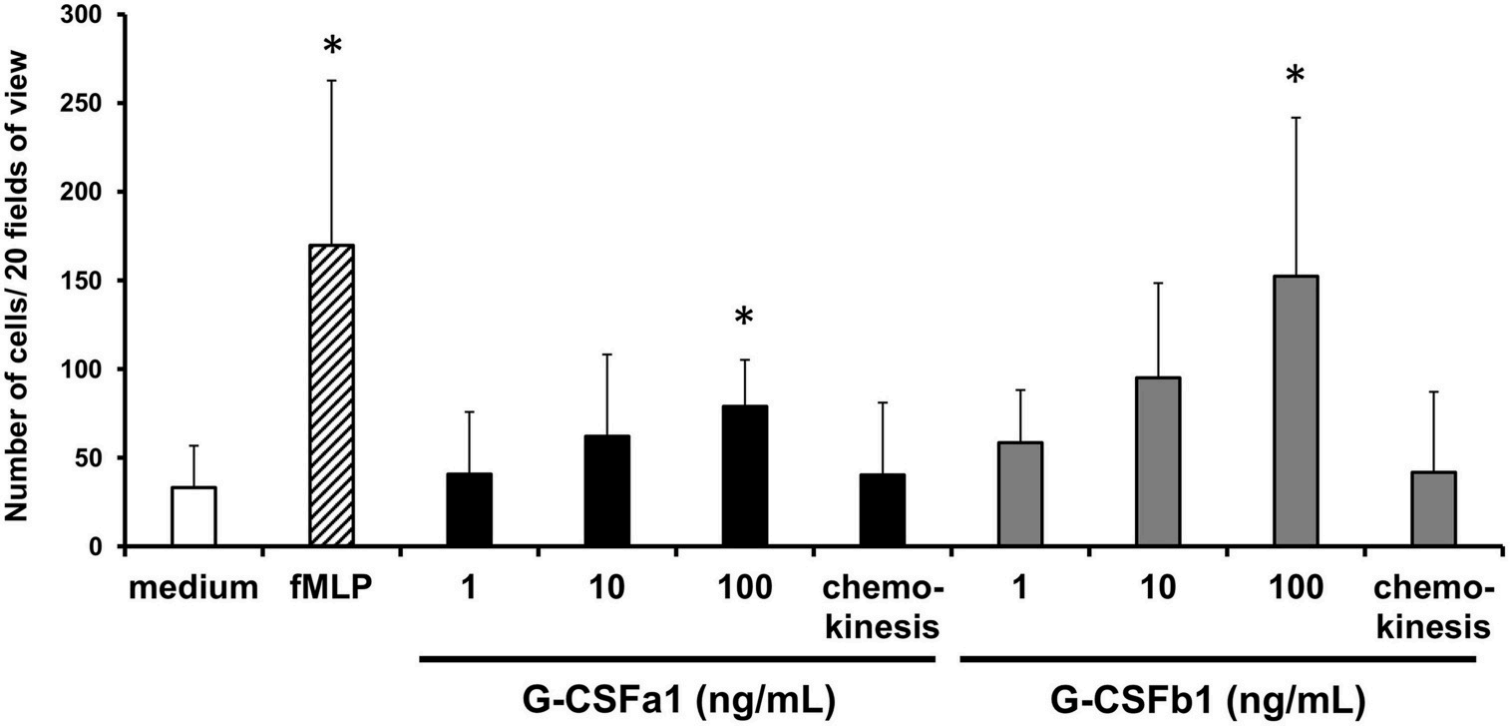
Recombinant G-CSFa1 and G-CSFb1 induces chemotactic response of kidney neutrophils. Chemotactic response of kidney neutrophils after 1 h of incubation with duplicate filters separating cells and cytokines at the concentrations indicated. Cells were stained with MGG and the total number of cells in 20 random fields of view (40× magnification) was determined. Medium and 10 ng/mL fMLP served as negative and positive controls, respectively. Equal concentrations (100 ng/mL) of cytokines in the upper and lower chemotaxis chambers served as chemokinesis control. The data represent mean + standard deviation (*n* = 4). Significant differences compared to medium control were determined using one-way ANOVA followed by Dunnet's *post-hoc* test, (*p* < 0.05) are denoted by asterisks (^*^).

Following the development of neutrophils at the sites of hematopoiesis, the migration and the recruitment of these cells toward the sites of infection or injury is essential for an efficient inflammatory response. We investigated the chemotactic effect of recombinant G-CSFa1 and G-CSFb1 on kidney neutrophils from normal adult carp employing a blind-well chemotaxis apparatus (Supplementary Figure S6). Neutrophils migrated toward fMLP placed in the bottom chamber (Figure 7), consistent with previous reports (22). In the presence of high doses of G-CSFa1 or G-CSFb1, kidney neutrophils migrated toward the sources (Figure 7). The chemokinesis controls indicated that neutrophil migration was cytokine-gradient dependent, since the migration of neutrophils was similar to the medium control when the recombinants were placed in both upper and lower chemotaxis chamber (Figure 7).

**Figure 8:**
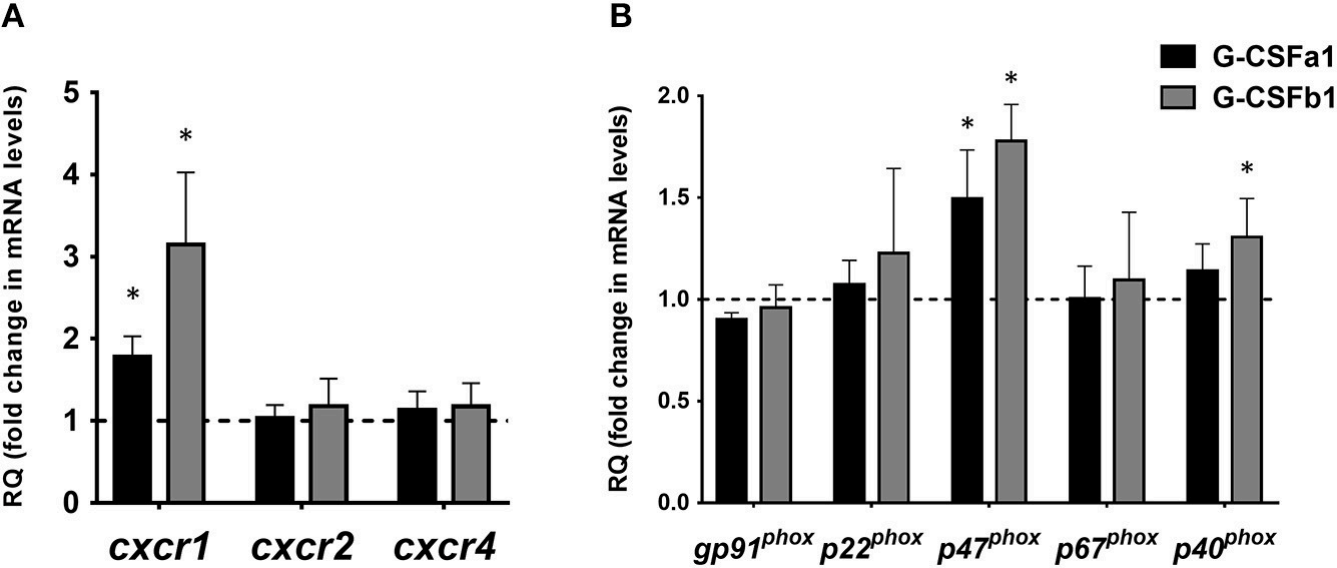
Recombinant G-CSFa1 and G-CSFb1 up-regulates *cxcr1* and *p47*^*phox*^ mRNA expression levels in carp kidney neutrophils. Quantitative expression analysis of mRNA levels of chemokine receptors **(A)** and NADPH oxidase components **(B)** in carp kidney neutrophils treated with the medium, 100 ng/mL G-CSFa1 and 100 ng/mL G-CSFb1 for 6 h. The mRNA levels were calculated using β*-actin* as a reference gene. Data were normalized to the control cells (dashed line at *y* = 1) and presented as mean + standard deviation (*n* = 3). Significant differences compared to unstimulated controls were determined using one-way ANOVA followed by Dunnet's *post-hoc* test, (*p* < 0.05) are denoted by asterisks (^*^).

To assess the ability of G-CSFa1 and G-CSFb1 to modulate the gene expression of chemokine receptors, kidney neutrophils were treated with medium, G-CSFa1 or G-CSFb1 for 6 h. Teleost CXCR1 and CXCR2 are conserved receptors for interleukin-8 (IL-8, also termed CXCL8) and are important for the regulation of neutrophil recruitment and migration to sites of infection and injury (44, 45). *Cxcr1* mRNA levels in neutrophils treated with G-CSFa1 and G-CSFb1 were significantly up-regulated compared to the medium control, indicating that both enhances a chemotactic sensibility of neutrophils toward chemotactic mediators such as IL-8 (Figure 8A). Neither *cxcr2* mRNA levels in neutrophils treated with G-CSFa1 nor G-CSFb1 were changed compared to the medium control (Figure 8A). The mRNA levels of *cxcr4* encoding a receptor for stromal cell-derived factor 1 (SDF-1, also termed CXCL12) in neutrophils were not modulated with the treatment of G-CSFa1 and G-CSFb1 (Figure 8A).

### G-CSFa1 and G-CSFb1 Enhance the Respiratory Burst Capacity in Kidney Neutrophils Through Up-Regulation of a NADPH Oxidase Component p47*^*phox*^*

The respiratory burst in neutrophils is the result of the formation of superoxide anions, in a process catalyzed by NADPH-oxidase (46, 47). Fish NADPH-oxidase components have been shown to have similar modes of activation and functional activities to mammalian counterparts (41, 48). To assess if the NADPH oxidase is induced by G-CSFa1 and G-CSFb1 treatments, we measured the mRNA levels of NADPH oxidase components (*gp91*^*phox*^, *p22*^*phox*^, *p47*^*phox*^, *p67*^*phox*^, and *p40*^*phox*^) in neutrophils treated with G-CSFa1 and G-CSFb1 for 6 h. mRNA levels of *p47*^*phox*^ in neutrophils treated with G-CSFa1 and G-CSFb1 and *p40*^*phox*^ in neutrophils treated with G-CSFb1 were significantly increased compared to the medium control. In contrast, mRNA levels of other components were not significantly changed (Figure 8B).

**Figure 9:**
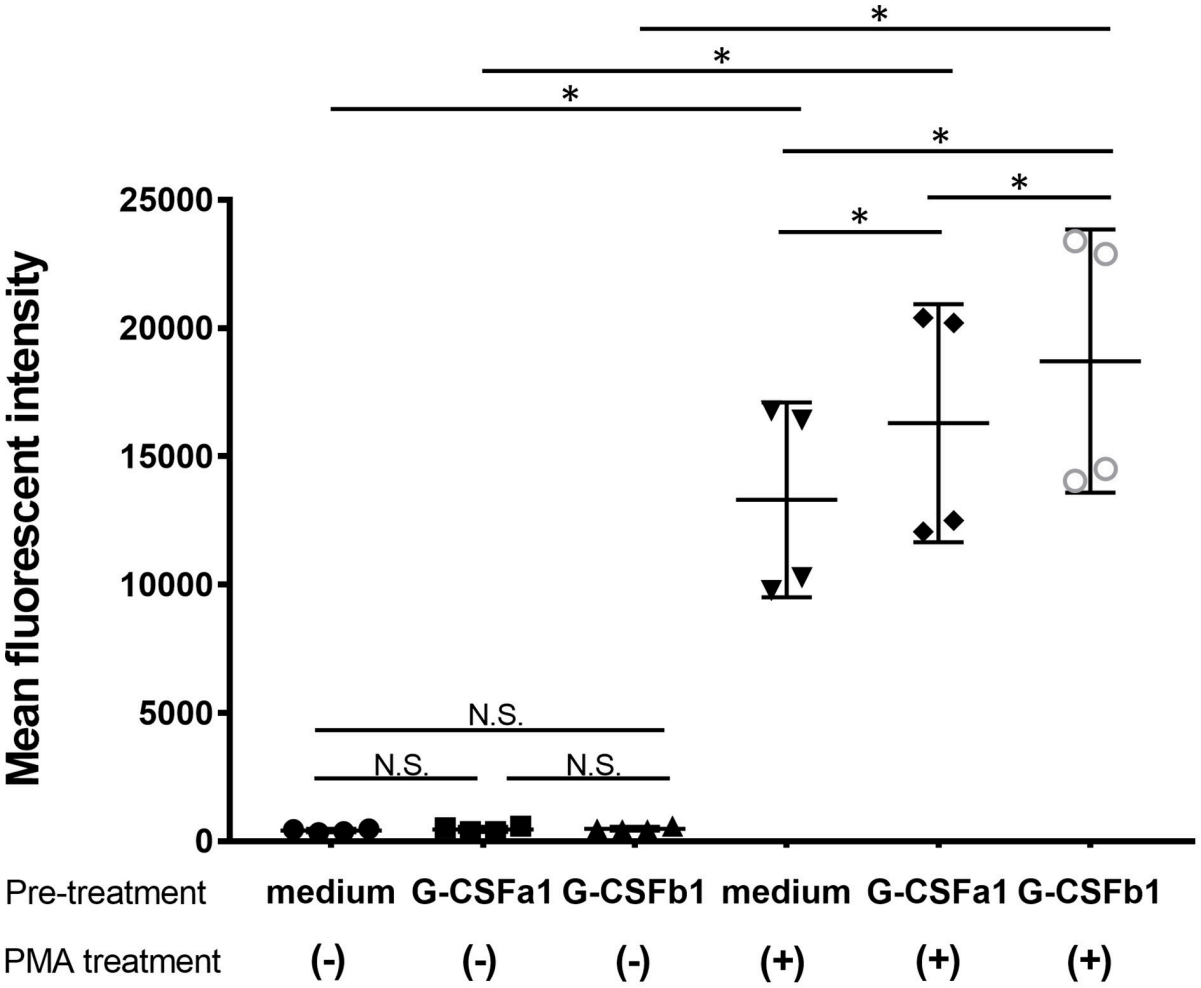
Recombinant G-CSFa1 and G-CSFb1 induces increased respiratory burst capability. Respiratory burst capability of kidney neutrophils after pre-treatment with the medium, 100 ng/mL G-CSFa1 or 100 ng/mL G-CSFb1 for 6 h and subsequently treated with or without 100 ng/mL PMA for 30 min in the presence of DHR123. Mean of DHR123 fluorescence intensity (MFI) in gated neutrophil population was measured by flow cytometry. Data points are presented as mean values of individuals and error bars show standard deviation. Kidney neutrophils were obtained from four fish. Significant differences compared to every other group with two factors of G-CSF pre-treatment and PMA treatment were determined using two-way ANOVA followed by Tukey's *post-hoc* test, (*p* < 0.05) are denoted by asterisks (^*^). N.S. represents ‘not significant’.

Furthermore, in order to investigate whether the treatment of carp kidney neutrophils with G-CSFa1 or G-CSFb1 induces their priming to prepare antimicrobial responses, we measured the respiratory burst in PMA-stimulated neutrophils. Neutrophils were pre-treated with the medium, G-CSFa1 or G-CSFb1 for 6 h. Following these treatments, neutrophils were treated with or without PMA in the presence of DHR123 and then analyzed by flow cytometry. Neither treatment of neutrophils with G-CSFa1 nor G-CSFb1 directly induced the respiratory burst without PMA stimulation (Figure 9). Both G-CSFa1 and G-CSFb1 significantly up-regulated the respiratory burst in PMA-stimulated neutrophils compared to the medium control, while the enhancement of respiratory burst in neutrophils treated with G-CSFb1 was higher than that of G-CSFa1 treated neutrophils (Figure 9), which is consistent with the result of the upregulation of *p47*^*phox*^ enhancement (Figure 8B).

### *In vivo* Administration of G-CSFa1 and G-CSFb1 Increases Circulating Neutrophils

**Figure 10:**
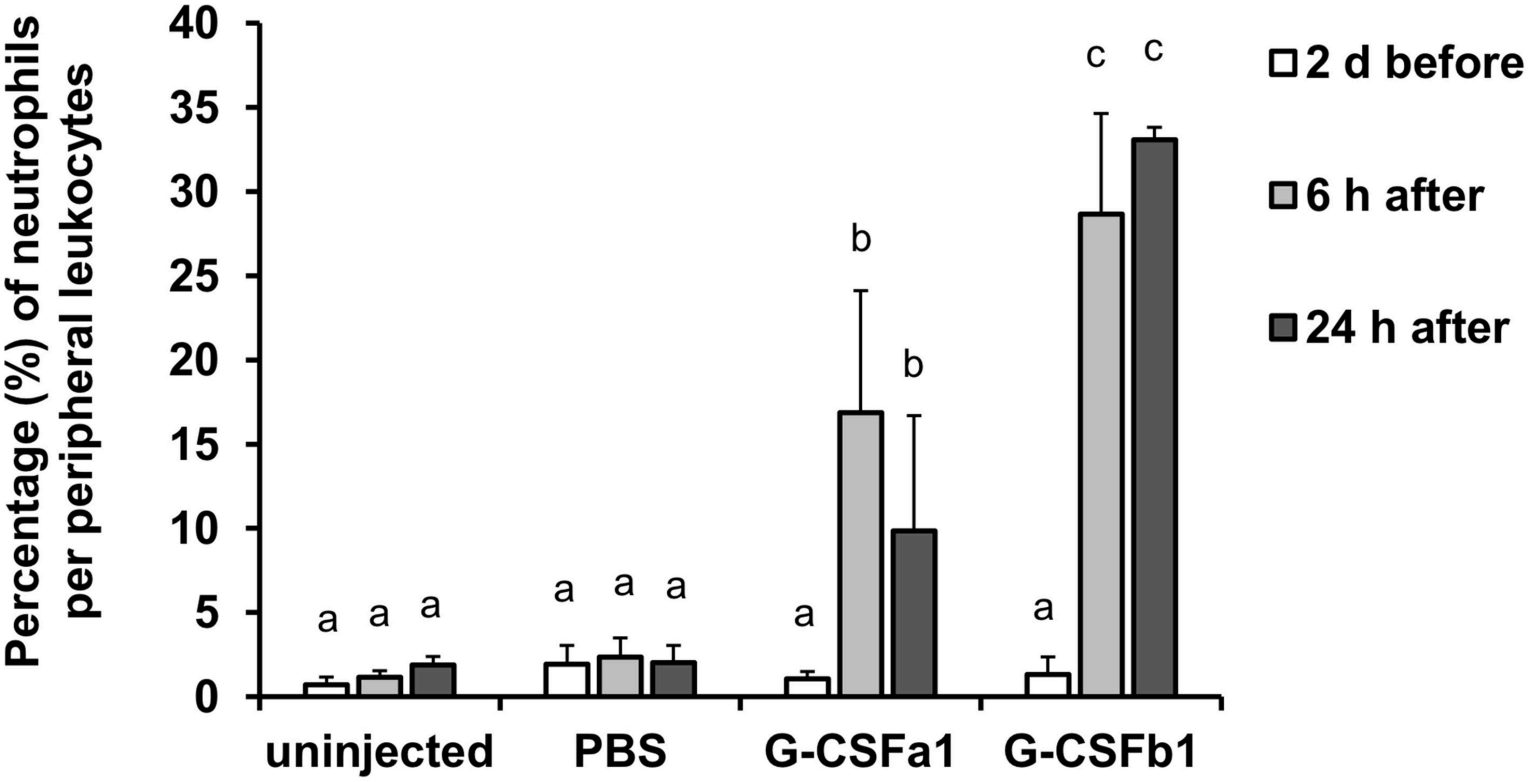
Administration of G-CSFa1 and G-CSFb1 to carp increases circulating blood neutrophil population. Peripheral blood leukocytes were collected from carp 2 days before and 6 and 24 h after intraperitoneal injection of 1 × PBS, recombinant G-CSFa1 and G-CSFb1 or of unhandled carp and analyzed by flow cytometry. Percentage of gated neutrophil population per live peripheral blood leukocytes was measured. Three fish for each group were used and data are presented as mean + standard deviation. Significant differences compared to other treatment groups and other time points were determined using two-way ANOVA followed by Tukey's *post-hoc* test, (*p* < 0.05) are denoted by different letters.

Following intraperitoneal (i.p.) injection of PBS and repeated bleeding, the population of peripheral blood neutrophils did not change for 24 h. In contrast, i.p. injection of G-CSFa1 induced a significant increase of peripheral blood neutrophils 6 and 24 h after injection. Likewise, the population of peripheral blood neutrophils was significantly increased after 6 and 24 h of G-CSFb1 injection. At 24 h, G-CSFb1 injection had induced a significantly higher circulating number of neutrophils than injection with G-CSFa1 (Figure 10). However, at 48 h after G-CSFa1 injection, neutrophil numbers no longer were higher than those of unhandled or PBS-injected fish, probably due to the repeated bleedings affecting the peripheral blood neutrophil population of the control groups (Supplementary Figure S7).

## Discussion

Here we cloned and functionally characterized carp G-CSF. All four carp *g-csf* genes contain five exons separated by four introns, retaining the gene structure found in human and mouse G-CSF as well as G-CSF of other teleost species (13). Carp and human G-CSF molecules share a similar structure of a signal peptide and a four-plus-one helical Pfam IL6/GCSF/MGF domain. All teleost fish G-CSF molecules share relatively high homology between each other at the helical regions. They also share acidic residues such as Asp and Glu, involved in the ligand-receptor binding, with mammalian G-CSF (49, 50). The four carp G-CSF paralogs identified in carp may have arisen from an ancestral G-CSF molecule through a series of duplications, including the teleost-specific 3rd WGD event and the carp-specific 4th WGD event (16, 21, 51–53). Overall, despite the overall low homology of teleost fish G-CSF sequences with mammalian G-CSF molecules, our *in-silico* analyses provide clear evidence that all four paralogs identified in carp are indeed orthologs of mammalian G-CSF.

Carp and other teleost fish G-CSF paralogs share only limited conservation of cysteine residues responsible for disulfide bonds with tetrapod G-CSF. Carp G-CSFa1 and G-CSFb1 express two structural differences: (i) an additional helix enriched with acidic residues in G-CSFa1 and (ii) a location of conserved cysteine residues. These structural differences prompted us to further investigate function of the different paralogs. Where *g-csfa1* and *g-csfa2* were highly expressed at basal level especially in spleen, *g-csfb1* and *g-csfb2* basal expression levels were very low in all tissues examined, indicating that g-csf transcription is differentially regulated between paralogs. Similarly, basal *g-csfa* gene expression was markedly higher than *g-csfb* expression in all immune cells examined. Macrophages are known to be the major cellular source of mammalian G-CSF (2). Strikingly, G-CSF paralogs were always highest expressed in macrophages of carp.

Basal gene expression levels of *g-csfa1* in macrophages were higher than those of *g-csfa2* and gene expression levels of *g-csfb1* in macrophages were higher than those of *g-csfb2*, which prompted us to further investigate function of G-CSFa1 and G-CSFb1 by production of these molecules as recombinant proteins. Recombinant proteins were produced in a bacterial expression system with the limitation that proteins are non-glycosylated and could possibly be contaminated with traces derived from bacteria. However, previous studies on mammalian G-CSF reported that glycosylation is not required for its activity and indeed, the non-glycosylated form is utilized in recombinant therapeutics (54). Even though the relative insensitivity to LPS has been reported in fish living in the aquatic environment with high pathogenic pressure (55), the recombinant proteins used in our assays were extensively purified up to the absence of LPS traces. Similar to mammalian G-CSF and zebrafish G-CSF (16, 56), carp G-CSF induced proliferation of hematopoietic cells in a dose-dependent manner, albeit with apparent different activities for the two paralogs studied: G-CSFa1 induced proliferation of blast-like cells adhesive to culture dishes, whereas G-CSFb1 induced proliferation of cells with neutrophil characteristics. Indeed, treatment with G-CSFb1 showed up-regulation of the transcription factor *cebp*α involved in neutrophil development (34). Also, we investigated at least two carp G-CSF receptor genes (data not shown) and found that only G-CSFb1 enhanced *gcsfr1* and *myeloperoxidase* (*mpx*) gene expression. Our data indicate that G-CSFb1 and G-CSFR1 are the main players involved with neutrophil development in carp. In zebrafish, both G-CSFa and G-CSFb may bind to the G-CSF receptor, expressed in both neutrophils and macrophages, and promote cell proliferation (16). In contrast to the latter study, carp G-CSFa1 alone did not stimulate colony formation in our semi-solid culture system, in which an agarose layer prevented natural formation of a stromal and an adherent cell layer. This possibly restricted access to spontaneously secreted growth factors from adherent macrophages (57), which are possibly required for colony formation. Further studies would be required to determine if G-CSFa1 directly induces macrophages to produce autocrine growth factors or that G-CSFa1 synergizes with some factors spontaneously secreted from adherent macrophages to synergistically stimulate macrophage development. Meanwhile, carp G-CSFb1 alone did stimulate CFU-G colony formation, whereas the combination of G-CSFa1 and G-CSFb1 stimulated formation of not only CFU-G but also CFU-GM colonies. Our data indicate that carp G-CSFb1 may drive granulopoiesis restricted to neutrophil-lineage development, whereas carp G-CSFa1 may be a cytokine with proliferative effect stimulating CFU-GM or earlier stem/progenitor cells. The functional differences between the G-CSFa1 and G-CSFb1 cytokine preparations make it highly unlikely that the induced cell responses could be due to traces of bacterial contaminations and thus appear cytokine-specific. No matter the indicative differences in biological function between paralogs, carp G-CSFs appears to act as a hematopoietic growth factors.

Mammalian G-CSF is chemo-attractive to neutrophils (58, 59). In zebrafish, G-CSFb but not G-CSFa could be linked to *in vivo* trafficking of neutrophils to the site of severe injury (19). Our results indicate that carp kidney neutrophils are strongly attracted to G-CSFb1 and are moderately attracted to G-CSFa1, possibly under influence of IL-8 (or CXCL8) (40, 60, 61). Indeed, treatment of carp kidney neutrophil with G-CSF paralogs showed a significant up-regulation of CXCR1 as the IL-8 receptor required for neutrophil recruitment, but not CXCR2 required for neutrophil reverse migration and resolution (45). Unlike mammalian G-CSF, carp G-CSF paralogs did not mediate transcription of CXCR4, important for retention of neutrophils in the hematopoietic tissue in mammalian models (62). In conclusion, carp G-CSFb appears to be the most important G-CSF paralog to induce neutrophil migration.

Once neutrophils receive inflammatory cytokine signals, they become “primed” and capable of promptly and vigorously exerting antimicrobial responses (63). We could not find a significant change of phagocytic activity in neutrophils against beads and zymosan particles following stimulation of G-CSF paralogs for any period tested (data not shown), indicating that neutrophil phagocytosis is regulated by other signals in fish. Although mammalian G-CSF alone is not able to initiate a respiratory burst in naïve neutrophils, pre-incubation with this cytokine primes the cell for an enhanced superoxide anion production following stimulation with physiological stimuli such as fMLP and PMA (11, 64). In fish, following stimulation of phagocytes with inflammatory cytokines, ROS production is activated through at least three sequential steps: (i) activation of protein kinase C (PKC), (ii) phosphorylation of p47^*phox*^, and (iii) the production of ROS catalyzed by the NADPH oxidase complex (48). In our hands, expression analysis of NADPH oxidase components in neutrophils treated with carp G-CSF paralogs exhibited up-regulation of especially p47^*phox*^, indicating that the priming effect of carp G-CSF paralogs on neutrophils was regulated through the increase of p47^*phox*^. Our study provides the first report in teleost fish on the priming effects of G-CSF on neutrophils and analysis of respiratory burst indicated that G-CSFb1 primed neutrophils more effectively than G-CSFa1.

Previous studies in cyprinids showed that circulating blood neutrophils increased in number 6 to 18 h after i.p. injection with killed *E. coli* or zymosan and then quickly decreased after 24 h, indicating that an intraperitoneal inflammation in fish induces a temporal mobilization of kidney-derived neutrophils into the circulation (42, 65). In our study, administration of G-CSF paralogs increased the number of circulating blood neutrophils 6 and 24 h after i.p. injection, suggesting that also *in vivo* G-CSFa1 and G-CSFb1 work as chemoattractants and granulopoietic growth factors, in agreement with the *in vitro* results. However, it remains unclear whether *in vivo* excess of G-CSF paralogs induce the expression of other inflammatory cytokines and/or chemokines in immune cells. In human clinical medicine, recombinant G-CSF is used as a biophylatic agent to specifically induce granulopoiesis in patients with chemotherapy- and radiation-induced neutropenia to prevent bacterial and fungal infections (66). Further studies will be required to investigate if fish G-CSF paralogs can act as biophylatic agents against infectious diseases in a similar way. Here, functional analyses were limited to G-CSFa1 and G-CSFb1, and we can only speculate that G-CSFa2 and G-CSFb2 could have similar, different, or combinatorial functions in common carp. Additional biochemical investigations involving all native carp G-CSF paralog proteins will be needed to elucidate the full and complicated picture of immune regulation in this polyploid species.

In summary, we identified four carp G-CSF paralogs, studied their gene expression patterns and characterized the functional differences between A and B types of G-CSF on carp hematopoietic cells and neutrophils. We report important differences in their regulation: A type G-CSFs have a relatively high constitutive gene expression and could thus be involved in maintenance of a homeostatic state, whereas B type G-CSFs have a low gene expression and require induction and could thus have a responsive, immunological role associated with a state of infection. In general, G-CSFa1 alone stimulates proliferation of granulocyte/macrophage progenitors, while G-CSFb1 promotes proliferation, differentiation and colony formation of granulocyte/macrophage progenitors and granulocyte progenitors in kidney of carp, similar to the G-CSF mammalian counterpart. G-CSFa1 and G-CSFb1 act as chemo-attractants to neutrophils modulating the expression of the chemokine receptor CXCR1, suggesting a role for G-CSF paralogs in neutrophil trafficking. Both, G-CSFa1 and G-CSFb1 appear to induce neutrophil “priming.” The carp G-CSF paralogs reported herein provide us with valuable tools to further study the immune system of teleost fish.

## Author Contributions

FK and KN: conceived and designed the experiments; FK, KN, AW, and EH: performed the experiments; FK, KN, AW, JM, and MO: analyzed the data; FK, KN, AW, GW, and TM: wrote and edited the paper.

### Conflict of Interest Statement

The authors declare that the research was conducted in the absence of any commercial or financial relationships that could be construed as a potential conflict of interest.

## Abbreviations

WGD: whole genome duplication
LPS: lipopolysaccharide
ConA: concanavaline A
PMA: phorbol 12-myristate 13-acetate
polyI:C: polyinosinic-polycytidylic acid
IPTG: isopropyl β-_D_-_L_-thiogalactopyranoside
MTT: 3-(4, 5-dimethyl-2-thiazolyl)-2, 5-diphenyltetrazolium bromide
CFU-G: granulocyte colony-forming unit
CFU-GM: granulocyte/macrophage colony-forming unit
fMLP: N-formyl-methionyl-leucyl-phenylalanine
DHR123: dihydrorhodamine 123
FITC: fluorescein isothiocyanate.
