# Supplementary material for: Paralogs of Common Carp Granulocyte Colony-Stimulating Factor (G-CSF) Have Different Functions Regarding Development, Trafficking and Activation of Neutrophils
Source: Front Immunol. 2019 Feb 19;10:255. doi: 10.3389/fimmu.2019.00255 (PMC6389648; doi:10.3389/fimmu.2019.00255)
Supplement: Supplementary file 1 [file Data_Sheet_1.PDF]

frontiers

Supplementary Material

Paralogs of common carp granulocyte colony-stimulating factor (G-CSF) have different functions with regard to development, trafficking and activation of neutrophils

Fumihiko Katakura[1, 4]*, Kohei Nishiya[1, 4], Annelieke S. Wentzel[2], Erika Hino[1], Jiro Miyamae[1], Masaharu Okano[1], Geert F. Wiegertjes[2, 3], Tadaaki Moritomo[1]

[1]Laboratory of Comparative Immunology, Department of Veterinary Medicine, Nihon University, Kameino 1866, Fujisawa, Kanagawa 252-0880, Japan

[2]Cell Biology and Immunology Group, [3]Aquaculture and Fisheries Group, Wageningen Institute of Animal Science, Wageningen University & Research, PO Box 338, 6700 AH, Wageningen, The Netherlands

[4]Authors with equal contribution

*** Correspondence:** Fumihiko Katakura, D.V.M., Ph.D.

Email: katakura.fumihiko@nihon-u.ac.jp

Supplementary table S1. Amino acid sequence identities (upper grids) and similarities (*lower grids*) of human, mouse, chicken, xenopus, and fish G-CSF orthologues. Amino acid identities and similarities (in percentages) were calculated using the Sequence Manipulation Suite v2: Ident and Sim (http://www.bioinformatics.org/sms2/ident_sim.html) following the multiple alignment with the Clustal Omega (https://www.ebi.ac.uk/Tools/msa/clustalo/) using default parameters. Accession numbers of protein sequences listed are same to Fig. 1B.

	Human	Mouse	Chicken	African-clawed frog	Zebrafish G-CSFa	Carp G-CSFa1	Carp G-CSFa2	Zebrafish G-CSFb	Carp G-CSFb1	Carp G-CSFb2
Human		69.95	35.21	22.86	16.07	14.40	13.88	18.64	20.81	21.82
Mouse	*76.06*		33.02	24.04	13.78	14.81	13.36	15.11	16.37	17.78
Chicken	*49.77*	*49.06*		25.37	14.29	15.25	14.71	15.28	18.89	19.35
African-clawed frog	*31.90*	*37.50*	*39.02*		15.81	13.25	13.56	13.62	13.55	14.55
Zebrafish G-CSFa	*29.91*	*29.78*	*30.41*	*29.77*		56.70	51.32	18.87	28.23	27.75
Carp G-CSFa1	*29.63*	*30.86*	*31.78*	*28.63*	*68.30*		69.74	18.61	28.07	28.95
Carp G-CSFa2	*27.35*	*28.34*	*30.67*	*26.69*	*60.96*	*75.44*		19.91	28.38	29.26
Zebrafish G-CSFb	*30.00*	*26.22*	*30.09*	*23.47*	*34.43*	*34.20*	*35.50*		34.34	36.36
Carp G-CSFb1	*37.10*	*34.51*	*36.87*	*27.10*	*44.98*	*44.30*	*44.54*	*53.54*		72.82
Carp G-CSFb2	*36.82*	*34.22*	*34.10*	*30.05*	*45.93*	*44.74*	*45.85*	*52.53*	*82.05*	

Supplementary table S2. Oligonucleotide primers used for cDNA cloning from carp and construction of expression plasmid vectors.

Primer name	Primer nucleotide sequences (5'-3')	Application
g-csfa1 F1	CACACTTGTGGGAATTGTGG	Partial sequencing
g-csfa1 R1	TACTGCGGAGGATGTCACAG	Partial sequencing
g-csfa1_3' F1	GCATCTGCCCCAATCTCTGACAAAC	3' RACE
g-csfa1_3' F2	GGCTCAGACAGACCAAGTAAAAGACC	3' RACE (nested PCR)
g-csfa1_5' R1	ACATGCAGGTTGACAAGCAA	5' RACE
g-csfa1_5' R2	TCTGATGCCTGCTTTGATGGGTCA	5' RACE (nested PCR)
g-csfa1_full F	ATGGGGACTGCTGCAATCTG	CDS cloning
g-csfa1_full R	CATGTATGCTAGGGTACAATGCTG	CDS cloning
g-csfa2_full F	CATGTTCTTTTTGATTACTGCTGTG	CDS cloning
g-csfa2_full R	AAGGGAGTTCCTAATGTGTTAAATTAA	CDS cloning
g-csfb1_3' F1	CGCGCCACGAGTTTAGCCAAGAAG	3' RACE
g-csfb1_3' F2	TCACACAGGACGTCTTCCGCAGTC	3' RACE (nested PCR)
g-csfb1_5' R1	AGACTGCGGAAGACGTCCTGTGTGAA	5' RACE
g-csfb1_5' R2	CGGCCACAATACGGCTTAAACTC	5' RACE (nested PCR)
g-csfb1_full F	CGCAATAACGAGACAGCTCA	CDS cloning
g-csfb1_full R	CACAGGGTACAACATCTGTCAA	CDS cloning
g-csfb2_full F	AGCCTGCTAGAAATCCCTTGA	CDS cloning
g-csfb2_full R	AAATATAGCAGGGACTGGTTGG	CDS cloning
G-CSFa1_ProExp_NdeI_F	CATATGGCCCCAATCTCTGA	Construction of an expression plasmid vector
G-CSFa1_ProExp_BamHI_R	GGATCCTCATAGACCTGCTTTA	Construction of an expression plasmid vector
G-CSFb1_ProExp_NdeI_F	CATATGGCGCCGCTCCAG	Construction of an expression plasmid vector
G-CSFb1_ProExp_BamHI_R	GGATCCCTAGTTTGATGCATCA	Construction of an expression plasmid vector

Supplementary table S3. Oligonucleotide primers used for gene expression analysis with quantitative RT-PCR.

Target (Accession No.)	Primer nucleotide sequences (5'-3')	Product length	Marker for
Carp g-csfa1 (MG882495)	F ACCCTCTGCCCCAGTTCTTC<br>R TCTGAGCCAGTGTGGTTGC	134 bp	G-CSFa1
Carp g-csfa2 (MG882496)	F TGGGCGACAACACGATTAGA<br>R TGAAGTTGCAGTCCCTTCACC	136 bp	G-CSFa2
Carp g-csfb1 (MG882497)	F TGAAGTTTTGCCTCATTCTTGC<br>R CAACGTCGCTCAGGATCTTCT	135 bp	G-CSFb1
Carp g-csfb2 (MG882498)	F CCACAGAATCCCAGAAAACCA<br>R GGCGTAGACTGCGGAAGACA	135 bp	G-CSFb2
Carp IL-1β (AJ245635)	F AAGGAGGCCAGTGGCTCTGT<br>R CCTGAAGAAGAGGAGGAGGCTGTCA	72 bp	Interleukin-1 beta
Carp cebpa (MH262559)	F AAGACACCGCTGGAGACCTG<br>R TTGCTTGGAGTTGTTGTGGAA	128 bp	Transcription factor (TF) involved in myelopoiesis
Carp pu.1 (XM_019107899)	F ACCGGGCATCACCTCACTCT<br>R CTGCTGGGGTCATCGTCATC	125 bp	TF involved in myelopoiesis
Carp irf8 (XM_019088951)	F ACTATGGAGGTCGGCTGGTG<br>R GATGTTCTGGAGGCTGTCTGG	122 bp	TF involved in myelopoiesis
Carp gata2 (AB429308)	F CCCATCCCAACCTACCCAAC<br>R TCACACATTCACGCCCCTCT	157 bp	TF involved in early hematopoiesis
Carp gata1 (AB429307)	F TGAGCCCTTCATCATTCTCC<br>R TCCGCAAGCATTACAGAGGT	146 bp	TF involved in erythropoiesis
Carp gata3 (AB429311)	F TATCGACGGACAGAGCAACC<br>R CCATCAAGCCAAGGAAGAGA	132 bp	TF involved in T lymphopoiesis
Carp pax5 (AB429310)	F CAGCGTCAGCTCAATCAACA<br>R TACTGCGGATACCTGGGTCA	121 bp	TF involved in B lymphopoiesis
Carp csf1r (AB526448)	F CAGGAAACCGGCCACTACA<br>R CCCATCTCACCATCGCTACA	106 bp	Colony-stimulating factor 1 receptor (monocyte/ macrophage)
Carp gcsfr1 (MH262557)	F TGTTCATACGATGGGTGGAAG<br>R AACACAGGCAAACACAACGA	145 bp	G-CSF receptor 1 (neutrophil)
Carp gcsfr2 (MH262558)	F GCTGGGCTCTGTCTCCTGTT<br>R CACTGAGGGATGCTGGTGTT	92 bp	G-CSF receptor 2
Carp mpx (AB429306)	F GTGGTCGTGTCGGTCCTCTT<br>R GATGCCTTTTGTTTGGTGGTG	118 bp	Myeloperoxidase (neutrophil)

Carp cxcr1 (AB010468)	F AGACGAATCACGCCGACATA<br>R GACCACCAGAGGGAAGAGGA	80 bp	Interleukin-8 receptor alpha subunit
Carp cxcr2 (AB010713)	F GCCATCGTGAAAGCAACC<br>R AGGCACATACCCACACCAAC	82 bp	Interleukin-8 receptor beta subunit
Carp cxcr4 (AB012310)	F TGACACGGGCATGAATACGA<br>R ATGTGCTGAAAGCGGAACAC	87 bp	Receptor for CXCL12
Carp gp91^{phox} (AB290328)	F TCATCAAGTGCCCATCCATC<br>R CCACGGTTTTGTCACCTCCA	150 bp	Transmembrane subunit of NADPH oxidase
Carp p22^{phox} (AB290329)	F TATGCGACTGGTGGGATTGT<br>R CTTTGCCCCGTTTGCTTC	124 bp	Transmembrane subunit of NADPH oxidase
Carp p47^{phox} (AB290330)	F GTGGGTGGTCAGGAAAGGAG<br>R GGGCGTTGCGTATGGTAGA	146 bp	Cytosolic subunit of NADPH oxidase
Carp p67^{phox} (AB290331)	F AGGCTCAGTTGGGAAAATGG<br>R TATGGCTGAACTTGGCGTCT	84 bp	Cytosolic subunit of NADPH oxidase
Carp p40^{phox} (AB290332)	F CCCCCACACGCAAAGTAAA<br>R CTGCCGCTGAAGTCAAACAC	88 bp	Cytosolic subunit of NADPH oxidase
Carp β-actin (M24113)	F CAAGGCCAACAGGGAAAAGA<br>R AGGCATACAGGGACAGCACA	98 bp	Internal control
Carp 40s (AB012087)	F CCGTGGGTGACATCGTTACA<br>R TCAGGACATTGAACCTCACTGTCT	69 bp	Internal control

Supplementary table S4. Oligonucleotide primers used for gene expression analysis with semi-quantitative RT-PCR.

Target (Accession No.)	Primer nucleotide sequences (5'-3')	Product length	Marker for
Carp gcsfr(1/2) (MH262557 / MH262558)	F GTTGTGTTTGCCTGTGTTGG<br>R CTGGTGGAGGGGATGAATG	379 bp	Neutrophil
Carp cebpa (MH262559)	F GCTGGAGACCTGAGCGAGAT<br>R GCGTGGTGTTGAGAGTGGTG	353 bp	Neutrophil
Carp mpx/mpo (AB429306)	F ACCACAGTATACCAGGCTATAATGC<br>R GGTTCTCAAACCATAACCTGTCC	270 bp	Neutrophil
Carp csf1r/mcsfr (AB526448)	F AACTAAAGCTCGGAAAGACTCTGG<br>R CGCAGGAAGTTCAGAAGATCAC	286 bp	Monocyte/ macrophage
Carp irf8 (XM_019088951)	F GTGCCAGAGGAGGAACAGAAG<br>R GATGTTCTGGAGGCTGTCTGG	414 bp	Monocyte/ macrophage
Carp lck (AB429309)	F CGTCGGGTGGCTATCAAGAG<br>R TGAGCTCATCGGACACCAAA	328 bp	T cell
Carp IgM heavy chain (ighm) (AB004105)	F TTCTTCCACCACCCCCAC<br>R GCTGCAATCTTGAATAGGAACTG	353 bp	B cell
Carp gata1 (AB429307)	F TTCCAGCTCTGAGACTGACTTACTGC<br>R CCCGTATGGACCCAGCATGT	442 bp	Erythrocyte
Carp β-actin (M24113)	F GTACGTTGCCATCCAGGCTGTG<br>R ACGTCACACTTCATGATGGAGTTGAAG	465 bp	Internal control

A

```
ACTGCTGCAATCTGGTGACTTTGCGCATTTTCTTTTTGATCACTGTTGCTATAATGAACTTCCAGGgt..(intron1;341
                                               M  N  F  Q  A
bp)..agCGGCTCTGTTAGTCACACTTGTGGGAATTGTGGCATCTGCCCCAATCTCTGACAAACTGGACATTATGAACAAGG
        A  L  L  V  T  L  V  G  I  V  A  S  A  P  I  S  D  K  L  D  I  M  N  K  D
ATACTATTGAACAGGCCCACAGTTTAATCAACAAAATTCTACAAGACATTCCTACAACTCATGCAGCCTGGATAAAGAGCAA
  T  I  E  Q  A  H  S  L  I  N  K  I  L  Q  D  I  P  T  T  H  A  A  W  I  K  S  K
Ggt..(intron2;769bp)..agAGTCTGACTTTGGGCGACAGCAAGGCTAGGCTGGAGTTGGAGTTCTTGAAGAAAGACA
                     S  L  T  L  G  D  S  K  A  R  L  E  L  E  F  L  K  K  D  M
TGTATATACCCTCTGCCCCAGTTCTTCAGCTCATTTCCAATAACTTCAGCATGgt..(intron3;228bp)..agGAAACC
  Y  I  P  S  A  P  V  L  Q  L  I  S  N  N  F  S  M                          E  T
TGCCTTGCAAACATAACGAAGGGACTGCAACTGCATCTCAATCTCTTGAAAGAGATCAGCAATGCAACCACACTGGCTCAGA
C  L  A  N  I  T  K  G  L  Q  L  H  L  N  L  L  K  E  I  S  N  A  T  T  L  A  Q  T
CAGACCAAGTAAAAGACCTTCAAGCTGAAATATATGAACTTCTGCTCCTAATTGAAGAGgt..(intron4;445bp)..ag
  D  Q  V  K  D  L  Q  A  E  I  Y  E  L  L  L  L  I  E  E
TTGCAGAACCAGGCAGGATTTGACCCATCAAAGCAGGCATCAGATGAGGAGTCGCAGACCCCTGAGCATGATCTGGCCAAGC
L  Q  N  Q  A  G  F  D  P  S  K  Q  A  S  D  E  E  S  Q  T  P  E  H  D  L  A  K  R
GTCTGACAAATGAATACCTGACCCAGGTGGCAGCCCACCTCACCCTGCAACAGCTTCAGGACTTCAGCTGTGACATCCTCCG
  L  T  N  E  Y  L  T  Q  V  A  A  H  L  T  L  Q  Q  L  Q  D  F  S  C  D  I  L  R
CAGTATTCACAGTATGACCTCCAGCTTGGCAGAGAACCCTAACACTGTGCAGCTCTGCGTAAATAAAGCAGGTCTATGAGCA
 S  I  H  S  M  T  S  S  L  A  E  N  P  N  T  V  Q  L  C  V  N  K  A  G  L  *
GCCTAGAGTGTAAAGCCCAAAGTATACCTGAGTTTTGACACAAACGCATTGCCTTTCAAAATAAACTTAAAGCCTGCAAAAT
TTACTGAAACACATGCAGGTTGACAAGCAAAACTGAAAGTTCCATCTTTGTCTAGTGTCTGTGCTATTCTTCCACAGATGCT
AGGAAAATTTGACTTCCCAATGGAAAAAAAAAAATAAACCTGGCTGTTGTACTCTATAGAGCATGCATAGATTAAACAGCATT
GTACCCTAGCATACATGTCAAAATCAAAGTAAACTTTGGGCTTTACACAAAAACAGATACAGTGACAAGTAAAAAACTAATT
AACAGCTGATATGTGTACACAGTCAAATTGTTTGTTGCTGGAATCTTGTTGCACCATCAGTCAGGACATGGAGGCAATGGTA
TTTGTTTCTTTGTATTTTTTCTTCAGATTTTGTATTTTTATTTATTTATTAATTAGGTTATTTATTTTATATTTCACTTGGC
ATTTTATTTATTATTTAAGTCATTTAGGAACTCCCCTAAATGATTGCATTCTTTACAGTCTCATAAAGTAATGTGTTACTTA
ATTCACTGTGAAATACTTTACTGTGTCATACAATCTGAATGCATGACCATGTCTCATAACTTGCCAAATGAGAACATGGTCA
TTGGATTGATCTCCTGATCTCTGTGAGAACTACACACTGTTTTTATTAGTCATTTCATGTGATATTATGAGATATGTAATGT
GATATCATACCCATAAAAAGTAAGTGAGATTTCAGATGAGTTTACGTATGAATCAGTATTATTTATTTATTTATTAATGTAA
CTAAAAATACATGTATTTATTTTTGCCATTATTTGTTATGATATTTTTGGAATATTTGAAGATGTGCCTTATGTTTGATCCA
AATAAACATTTCTGATGTGTGTTAAA
```

Supplementary figure S1A

Supplementary figure S1. Genomic sequences encoding carp G-CSF paralogs. (A) Genomic sequences encoding carp G-CSFa1. LHQP01022752.1, *Cyprinus carpio* isolate UL-001 Contig22774.

B

```
GCTTTGTTACTGCTGGGGTCTGGTGACTTTGTGCATGTTCTTTTTGATTACTGCTGTGATAATGAACTTCCAGGgt..(int
                                                              M  N  F  Q  A
ron1;359bp)..agCGGCTATATTAGTCACACTTGTGGGAATTGTGGCATCTGCCCCAATCTCTGACAAACTGGACATTTT
                  A  I  L  V  T  L  V  G  I  V  A  S  A  P  I  S  D  K  L  D  I  L
GAACAAGGATACTATTGAACAAGCCCACAGTTTAATCAACAAAATTTTAGAAGATGCTCCTAAAGCTCATGCAGCCTGGATA
 N  K  D  T  I  E  Q  A  H  S  L  I  N  K  I  L  E  D  A  P  K  A  H  A  A  W  I
AATAACAAGgt..(intron2;477bp)..agGGTCTGACTTTGGGCGACAACACGATTAGACTGCAGCTGAATTACTTGAA
N  N  K                           G  L  T  L  G  D  N  T  I  R  L  Q  L  N  Y  L  K
ATCTGTGATACCCTCTGCCCCAGTGCTTCAGAACATCTCCAATATCTCCAGCATGgt..(intron3;502bp)..agGAAA
 S  V  I  P  S  A  P  V  L  Q  N  I  S  N  I  S  S  M                          E  T
CCTGCCTTGCAGACATGGTGAAGGGACTGCAACTTCATCTGAATCTCCTGAATGAGATCATCAAAAAACTGGCCCAGACAGA
  C  L  A  D  M  V  K  G  L  Q  L  H  L  N  L  L  N  E  I  I  K  K  L  A  Q  T  D
CCAAGTGAATGTACTTAAATCTGAAATTCAAGAACTTCATTCCCTAATCAAAAAGgt..(intron4;369bp)..agTTGC
 Q  V  N  V  L  K  S  E  I  Q  E  L  H  S  L  I  K  K                          L  Q
AGAAACAGGCGGGATTTGACCCATCAAAGCATGCAAAAGATGAGCAGTCACAGACCCTTGTGCATGATCTGCACAAGCATCT
  K  Q  A  G  F  D  P  S  K  H  A  K  D  E  Q  S  Q  T  L  V  H  D  L  H  K  H  L
GACAACGGAATTCATGATCCAGGTGGCCGCCCACCTCACCCTGCAACAGCTTCAGGACTTCAGCTGTGACGTCCTCTGCAGT
 T  T  E  F  M  I  Q  V  A  A  H  L  T  L  Q  Q  L  Q  D  F  S  C  D  V  L  C  S
TTTCTTAGTATTCGCAGAATGACCTCCAACATGTCCATGGCAGAGAACCCTAAGACTGTGCAGCTCTGCATAAATGCAGCAG
F  L  S  I  R  R  M  T  S  N  M  S  M  A  E  N  P  K  T  V  Q  L  C  I  N  A  A  G
GTCTATGAGCAGCGTAGACTGTTAAGCCCAAAGTATACTTGAGTTTTGACACAGAAGCA
  L  *
```

Supplementary figure S1B

Supplementary figure S1. Genomic sequences encoding carp G-CSF paralogs. (B) Genomic sequences encoding carp G-CSFa2. LHQP01015831.1, *Cyprinus carpio* isolate UL-001 Contig15846.

C

GTGACGCAGCGCTTACGGATGCGGTCTGGGCTTTTTCTCGCTGTTCCTGCAGTGACGAAACAGAAGAATTCCCGGGAGATTT
CTCCTTCAGACCCGTCAGATCCGGACCCGAGGGATATTAAACATCTATGACCATACCATTAAATTCGCCCGGAAAATAAGGG
GCATTTTGCTGCGGAGCACTTGCTTTGATTCACACAAGAGCAGAACTAAGTTCAAGAAACGTGAAATAATTTCCACTTTTTG
GGGCGTTTTCACTCCCCCCCCCCCAAAGAACAGGAGACACAGGAGTGTGTTTTTCTCCTTGCACCTTTCCTCACTGGTTTTT
CGCAACGCAGCGTACACGTAATAGATTATAATGTAGTCGCAATAACGAGACAGCTCATCAAAGTTTCCTCAGTTGGACAATC
TCAATGGGTTTAAATAGGCTACTAGAATGCAGTAAAATATTCGATACACTCACAACCACA**ATGAAGTTTTGTCTCA**gt..(I
M K F C L I
ntron1;140bp)..ag**TTCTTGCAGTGTTGCGCTGCTGTCTGACGCTGGTGGACGCCGCGCCGCTCCAGAAGCAGGAGATG**
L A V L R C C L T L V D A A P L Q K Q E M
ACGCGCGCTGTGGAGCGCGCCACGAGTTTAGCCAAGAAGATCCTGAGCGACGTTCCCGCGGCGCACCAGGCGTGCGTCAACA
T R A V E R A T S L A K K I L S D V P A A H Q A C V N T
CCGCGgt..(intron2;220bp)..ag**GGTTTGGCTCTCTCCAGCGAAGCAGGACACTTGGAGTATTTCTTAAGTGACCTT**
A G L A L S S E A G H L E Y F L S D L
GGCATCCCCGCGCCGCCGGTGCTCAAGTCAGAGGACCTCAGCATGgt..(intron3;420bp)..ag**GACGTGAGTTTAAG**
G I P A P P V L K S E D L S M D V S L S
CCGTATTGTGGCCGGTCTGGACCTACACCGTGACCTCCTGCAGGACATCCGAGAGCGCTCGAGCTCCACAGAAGAACTGAGC
R I V A G L D L H R D L L Q D I R E R S S S T E E L S
CTCCTGCTCGCTGATATCACAGACCTGTCTGCTCAGGTCCACCAGgt..(intron4;98bp)..ag**ATGCAGCAGCTGGCC**
L L L A D I T D L S A Q V H Q M Q Q L A
CAGATTCCCAGCACAGTGTCCCAGAAGGCAGCGTTCCCAGCGCTCTCTCCACGGCTCAGCGGTGATTATCAAGTCCAAGTGG
Q I P S T V S Q K A A F P A L S P R L S G D Y Q V Q V A
CCATCCATCTTTCTCTTCAGCAGCTGCGCAGCTTCACACAGGACGTCTTCCGCAGTCTGCGCCACATTGCTGCATCAAACTA
I H L S L Q Q L R S F T Q D V F R S L R H I A A S N *
GCTGACTTTTCTGTGTGGAGTGTATTTTTGTAAGGCTCACTGGTTGTAAAACATTTGAATGGGTTATCCTTGCTAAGTTTGA
GCTGAACTTTGGAATAACTgttgttttaaatggatgtTTTTTATGGTAATTATTTAAAGTATTTATTGTTTATTTAATATAT
AACTTAAATATTTTTTATTGCTGATTTTATTTTTATGATGTTATATTGACAGATGTTGTACCCTGTGGGTACTGTGAAGATA
TCTATTAAACCTTATTTATTACTGA

Supplementary figure S1C

Supplementary figure S1. Genomic sequences encoding carp G-CSF paralogs. (C) Genomic sequences encoding carp G-CSFb1. LHQP01012478.1, *Cyprinus carpio* isolate UL-001 Contig12486.

D

TCCGCACGTGTGTGTCAGACAGCAGTTAAACACTACATCATCACATTAATCATCACATTAGTAAAGGGCTTTTCGCTGATGA

TCGCTTGCTCTGATTCGCGCAAGAGCAGAACTGAGTACAAGAAACGTGAAATAAGGTTTTGGGGCGTTCTCACTTTCCCCCC

CACATTTAAAGAACCGGAAACATAGGAGTGTCGTTTTCTCTTTTTTCCCCTATTGGTTTTTCGCAATGCATTGTACGTAATA

GGACCACAGTTTAGGTGAAATAGCTAGACAACTCATGGAAGTTTCCCCAGTTGAACAATCTGAACGGGTTTAAATAGCCTGC

TAGAAATCCCTTGATCAATGCAGTCAAATATCCCAAACTCTGTGAAGCACA**ATGCAGCTTTACCTCA**gt..(intron1;26

M Q L Y L I

5bp)..ag**TTCTCGCAGCGCACTGCTGTCTGACGCTGGTGGACTCCGCGCCGCTCCGGACGCACGAGCTGACGCGCGCCGTG**

L A A H C C L T L V D S A P L R T H E L T R A V

GACAGCGCCATGAGTTTAGCCAGGAAGATCCTGAGCGACATTCCCGCAGCGCACGAGGCGTGCGTCAAAGCCACGgt..(in

D S A M S L A R K I L S D I P A A H E A C V K A T

tron2;167bp)..ag**GGTTTGACCCTTTCCAGTGAATCAGAACACTTGGAGTATTTACTGAGTGACATTGGCATCCCTGCG**

G L T L S S E S E H L E Y L L S D I G I P A

CCTCCGCTGCTCAAGTCAGAGCACCTCAGCCTGgt..(intron3;1591bp)..ag**GATGTGAGTTTAAGCCGTATTGTAG**

P P L L K S E H L S L D V S L S R I V D

ACGGTTTGGAGCTACACCACAAACTTCTGCAGGAAATTAAAGAGCTCTTGACCTCCACAGAAGAACTGACCCTGCTGCTGGC

G L E L H H K L L Q E I K E L L T S T E E L T L L L A

AGATATTACAGACCTGTCTGCTCAGGTCCATAAGgt..(intron4;88bp)..ag**ATGCAGCAGCTGGCCCAGATTCCCAC**

D I T D L S A Q V H K M Q Q L A Q I P T

AGAATCCCAGAAAACCACGTTCCCAGCGATCTCTCCACAGCTCAGCAGTGACTATCATGTCCAAGTGGCCATCCACCTTTGT

E S Q K T T F P A I S P Q L S S D Y H V Q V A I H L C

CTTCAGCAGCTGCGCAGTTTTACTCATGATGTCTTCCGCAGTCTACGCCACATTGCTGCATCAAACTAGTTGACCAGTACTA

L Q Q L R S F T H D V F R S L R H I A A S N *

ACCCAACCAACCAGTCCCTGCTATATTTTGTATTTTCTGCGTAGAAATGTATTTTTGTAATGCTCACTGTTTGTGTTATCCT

TGCTAAGTTTAAGATGAACTTTAGAATAATTTTATTTAATCAGATGTTTTAATTTTAATGTATTTAATATTTA

Supplementary figure S1D

Supplementary figure S1. Genomic sequences encoding carp G-CSF paralogs. (D) Genomic sequences encoding carp G-CSFb2. LHQP01028996.1, *Cyprinus carpio* isolate UL-001 Contig29026, reverse complement.

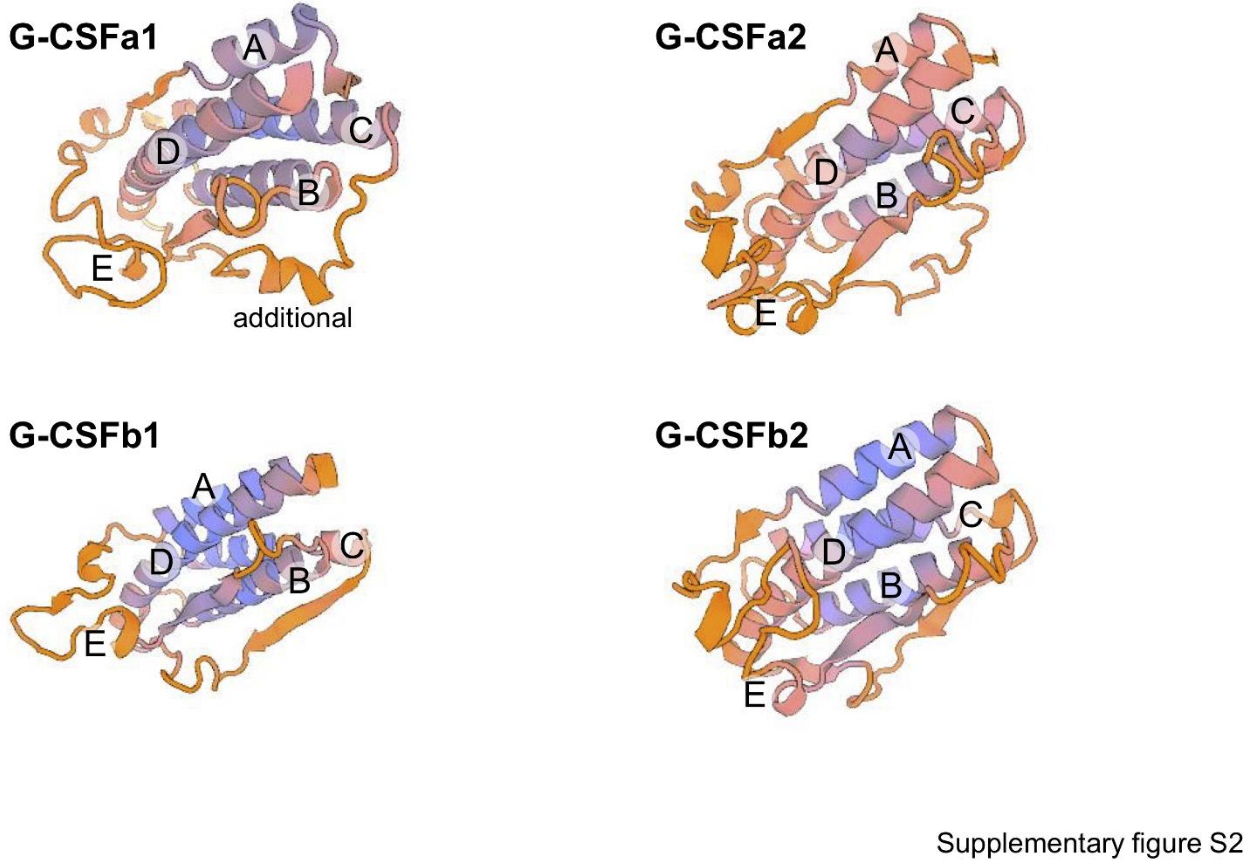

Supplementary figure S2. Proposed 3D structure of carp G-CSFa1, G-CSFa2, G-CSFb1 and G-CSFb2. Putative structures are modeled based on the structure of human G-CSF using the SWISS-MODEL server (https://swissmodel.expasy.org/).

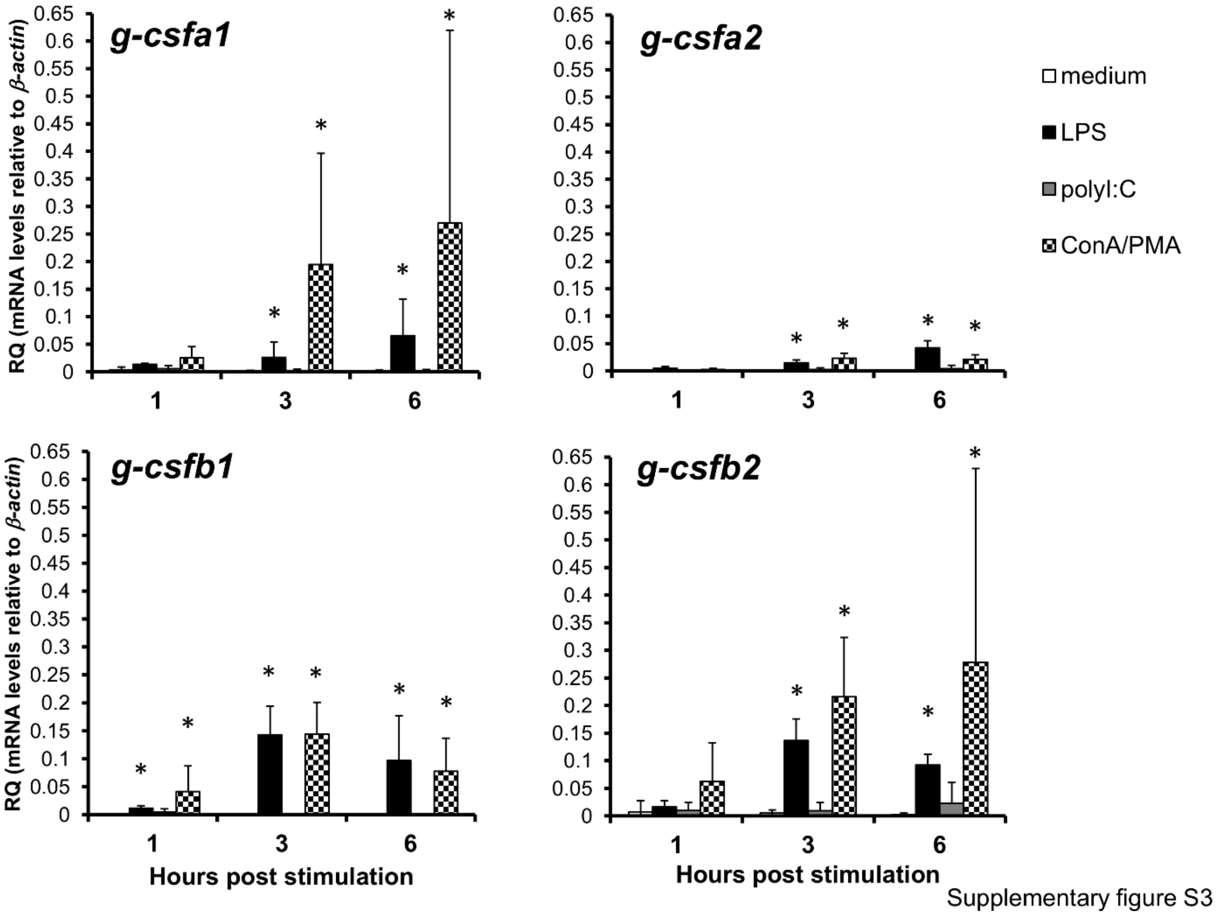

Supplementary figure S3. Quantitative mRNA expression analysis of four carp G-CSF paralogs in kidney leukocytes stimulated with mitogens for 1, 3 and 6 h. Freshly isolated kidney leukocytes from normal carp were treated with the medium, 50 µg/mL LPS, 50 µg/mL polyI:C, or a combination of 10 µg/mL ConA and 1 µg/mL PMA for 1, 3 and 6 h. The relative mRNA levels were calculated using *β-actin* as reference gene. Data represent mean + standard deviation ($n = 3$). Significant differences compared to the reference sample were determined using one-way ANOVA followed by Dunnet's post hoc test, ($p<0.05$) is denoted by (*).

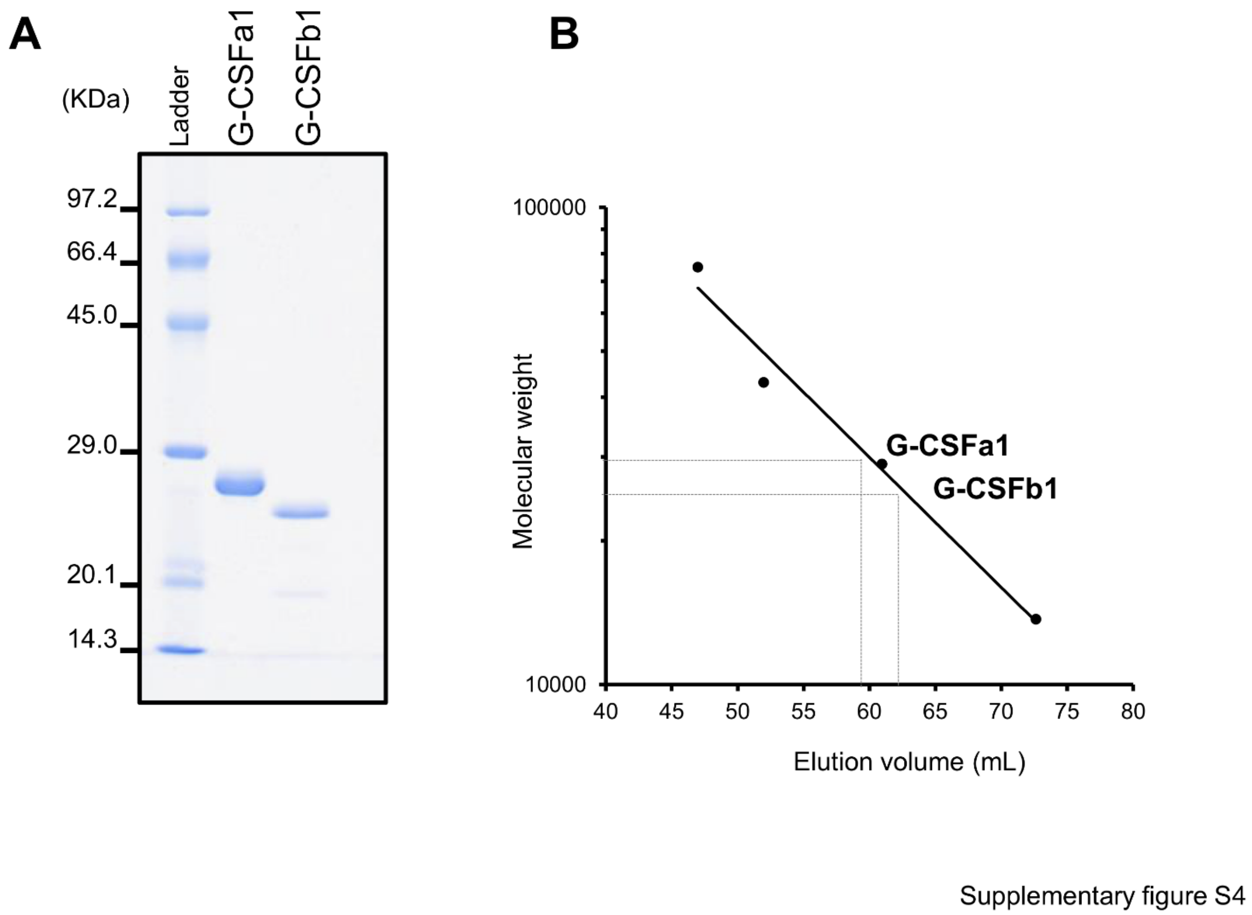

Supplementary figure S4. Purification and characterization of recombinant carp G-CSFa1 and G-CSFb1. (A) Purified G-CSFa1 and G-CSFb1 were separated on a 12.5% SDS-polyacrylamide gel under reducing conditions and visualized by staining with Coomassie Brilliant Blue R-250. (B) Molecular weight of the recombinant proteins were determined with gel filtration chromatography using a Sephacryl S-100 column (HR 16/60) under native condition. The molecular weights of the standard proteins: conalbumin, 75,000; ovalbumin, 44,000; carbonic anhydrase, 29,000; ribonuclease A, 13,700.

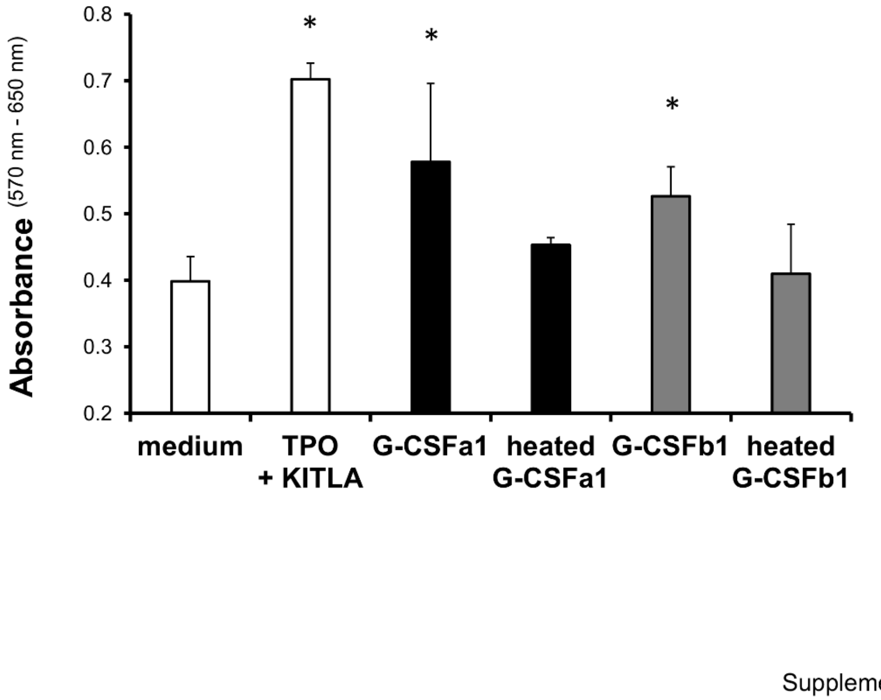

Supplementary figure S5. Proliferation of carp kidney neutrophilic granulocyte-like cells. Proliferative response of carp kidney leukocytes (40,000 cells) treated with medium alone, recombinant carp TPO (100 ng/mL) plus KITLA (100 ng/mL), recombinant carp G-CSFa1 (100 ng/mL), G-CSFb1 (100 ng/mL) or heat-inactivated G-CSF paralogs. Live cells treated with different stimuli were measured with the MTT assay at day 6 in the culture. Absorbance values at 650 nm were subtracted from experimental absorbance values at 570 nm in each well. Data represent mean + standard deviation ($n = 3$). Significant differences compared to the reference (medium) group were determined using one-way ANOVA followed by Dunnet's post hoc test, (p<0.05) is denoted by (*).

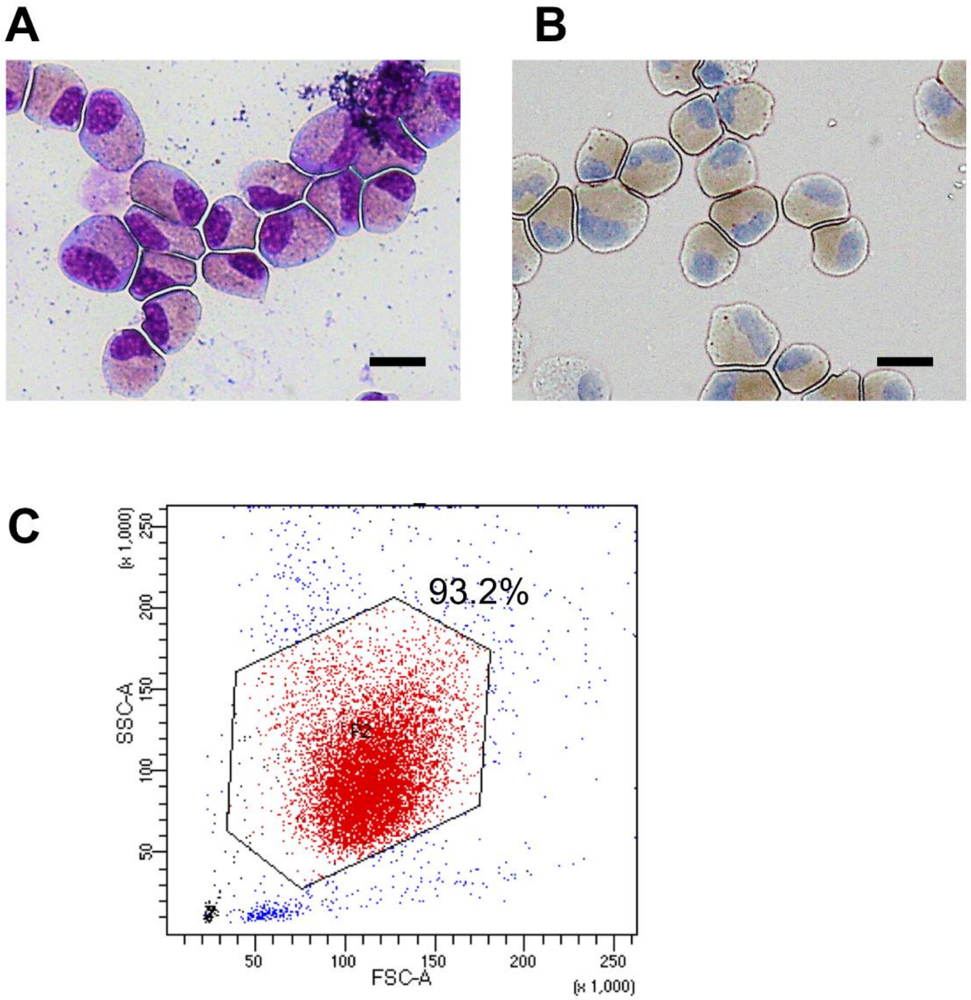

Supplementary figure S6

Supplementary figure S6. Isolation of kidney neutrophils from carp. (A, B) May-Grunwald Giemsa staining (A) and Peroxidase staining (B) of isolated kidney neutrophils. Bars indicate 10 µm. (C) Flow cytometric profile of isolated kidney neutrophils.

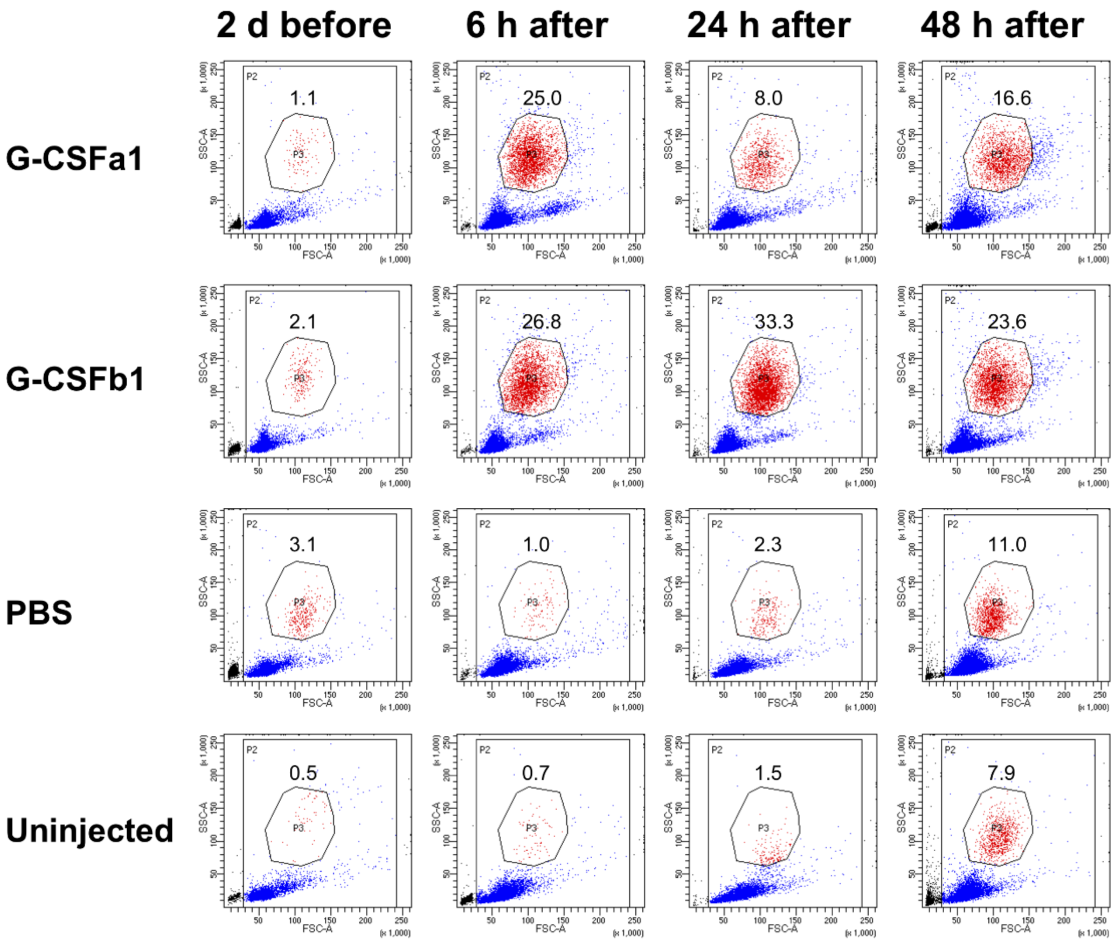

Supplementary figure S7

Supplementary figure S7. Flow cytometry analysis of peripheral blood leukocytes from carp i.p. injected recombinant G-CSFa1, G-CSFb1 and PBS and uninjected. Peripheral blood leukocytes were collected over time from carp intraperitoneally injected with 1xPBS, recombinant G-CSFa1 and G-CSFb1 and uninjected. Ten thousand leukocytes were analyzed by flow cytometry based on the depiction in forward scatter and side scatter parameters. P3 gates represent the neutrophil population with high side scatter and numbers show percentages of neutrophils in PI$^-$ live cells. Representative data in three fish per group are shown.
